# Enhanced DWT for Denoising Heartbeat Signal in Non-Invasive Detection

**DOI:** 10.3390/s25061743

**Published:** 2025-03-11

**Authors:** Peibin Zhu, Lei Feng, Kaimin Yu, Yuanfang Zhang, Wen Chen, Jianzhong Hao

**Affiliations:** 1 School of Ocean Information Engineering, Jimei University, Xiamen 361021, China; peibin.zhu@jmu.edu.cn (P.Z.); 202211810001@jmu.edu.cn (L.F.); 202311810006@jmu.edu.cn (Y.Z.); 2 School of Marine Equipment and Mechanical Engineering, Jimei University, Xiamen 361021, China; 202212855006@jmu.edu.cn; 3 Institute for Infocomm Research (I2R), Agency for Science, Technology and Research (A*STAR), Singapore 138632, Singapore; haoemily@i2r.a-star.edu.sg

**Keywords:** heart impact signal, ACF, enhanced threshold function, fiber optic microvibration, de-noising

## Abstract

Achieving both accurate and real-time monitoring heartbeat signals by non-invasive sensing techniques is challenging due to various noise interferences. In this paper, we propose an enhanced discrete wavelet transform (DWT) method that incorporates objective denoising quality assessment metrics to determine accurate thresholds and adaptive threshold functions. Our approach begins by denoising ECG signals from various databases, introducing several types of typical noise, including additive white Gaussian (AWG) noise, baseline wandering noise, electrode motion noise, and muscle artifacts. The results show that for Gaussian white noise denoising, the enhanced DWT can achieve 1–5 dB SNR improvement compared to the traditional DWT method, while for real noise denoising, our proposed method improves the SNR tens or even hundreds of times that of the state-of-the-art denoising techniques. Furthermore, we validate the effectiveness of the enhanced DWT method by visualizing and comparing the denoising results of heartbeat signals monitored by fiber-optic micro-vibration sensors against those obtained using other denoising methods. The improved DWT enhances the quality of heartbeat signals from non-invasive sensors, thereby increasing the accuracy of cardiovascular disease diagnosis.

## 1. Introduction

Non-invasive real-time monitoring of respiration and heart rate, such as optical heart rate monitoring of blood flow on the skin surface, heart rate accelerometers, millimeter wave radar, or camera-based heart rate monitoring, plays a crucial role in the early detection of hidden cardiovascular diseases and in providing effective health management [1,2,3,4,5,6,7,8]. However, the signals obtained from these techniques are often subject to various noises and artifacts [9,10,11,12,13]. Therefore, it is essential to perform noise reduction before analyzing and diagnosing cardiovascular conditions [14,15,16]. Various methods have been developed to suppress noise in cardiac signal processing, broadly categorized into classical signal processing frameworks and artificial intelligence (AI) methods. While conventional algorithms have computational efficiency suitable for real-time applications, their performance in removing noise is often limited by noise estimation mechanisms. On the other hand, while AI-based approaches can achieve excellent noise suppression with sufficient training data, they also face significant challenges. Deep learning models rely heavily on extensive annotated datasets; however, acquiring and annotating such data is both labor-intensive and time-consuming, especially for specialized or rare signals. In addition, training deep neural networks requires significant computational power, often requiring high-performance GPUs or even distributed computing resources, leading to increased resource consumption. At the same time, AI methods are less adaptive to environmental changes and usually need to be retrained or tuned when signal sources or noise conditions change, increasing application complexity. Hence, enhancing traditional denoising methods or their combinations, such as Empirical Mode Decomposition (EMD), Singular Spectrum Analysis (SSA), Variable Mode Decomposition (VMD) and Non-Localized Mean Filtering (NLM), presents a practical solution [17,18,19,20]. However, each of these traditional denoising techniques has its own advantages and disadvantages. For example, EMD is an adaptive technique that decomposes signals without predefined basis functions, making it effective for nonlinear and non-smooth signals. However, it is susceptible to mode aliasing, where signal components of different time scales are misclassified into the same intrinsic mode function (IMF), compromising decomposition accuracy. Moreover, its iterative process and dependence on local extrema detection incur high computational costs, posing challenges for real-time ECG processing and limiting its practicality in real-time monitoring systems [21]. EEMD enhances EMD by mitigating mode aliasing through the addition of white noise. By decomposing the signal multiple times with different noise sequences and averaging the results, EEMD improves decomposition stability. However, this approach significantly increases computational complexity, making it less suitable for real-time applications with limited resources. Additionally, the injected noise, while aiding decomposition, may introduce errors, potentially affecting the accuracy of the results [22]. Complementary EEMD (CEEMD) enhances EMD by improving decomposition robustness and reducing pseudo-modal effects. However, it remains sensitive to endpoint effects and requires the careful selection of noise amplitude and ensemble averages, which impact denoising performance [23]. VMD decomposes signals into bandwidth-limited modes through variational optimization, making it effective for non-smooth signals. However, its performance depends on parameter selection, and its high computational cost limits real-time applicability [24]. In contrast, Wavelet Transform (WT) is effective for multi-scale analysis and denoising non-stationary heartbeat signals due to its sparsity and efficiency. It is commonly applied in fields such as image processing, audio analysis, video processing, and mechanical systems [25]. Various types of WTs, such as Spcshrink’s Discrete WT (SDWT), Stationary WT (SWT), Stationary Wavelet Packet Transform (SWPT), and Dual Tree Complex Wavelet Transform (DTCWT), have been developed to cater to different needs [26,27,28,29]. Despite the versatility of WT methods, effectively removing noise from signals remains challenging due to the difficulty in accurately determining thresholds with suitable threshold functions. Typically, thresholds and threshold functions rely on noise levels or signal characteristics [30,31,32,33,34,35,36,37]. Current threshold functions struggle to eliminate pseudo Gibbs phenomena and constant biases effectively [38,39,40,41,42,43], regardless of whether they utilize specific functions or combinations of soft and hard thresholds [44,45,46,47,48,49,50,51,52]. Moreover, insufficient attention has been given to optimizing the wavelet decomposition level in recent studies [53,54]. In summary, WT-based methods encounter challenges in threshold selection, threshold functions, decomposition levels, and wavelet bases, which limit their effectiveness and influence the denoising performance of wavelet-based and hybrid filtering approaches [55,56,57,58].

The aim of this paper is to improve the wavelet denoising quality of heartbeat signals monitored in real time by non-invasive devices. First, an objective evaluation metric [36] is introduced to assess the denoising quality so as to realize the exact threshold and adaptive threshold function. Second, in order to quantitatively evaluate the denoising quality, a relatively clean ECG signal with added AWG noise is selected to verify the proposed exact threshold and adaptive threshold since the SNR and RMSE can be accurately calculated. The proposed method is validated by comparing it with existing wavelet methods to verify its effectiveness. Next, the method is applied to denoise six heartbeat signals from three different datasets, each contaminated with three common types of noise: baseline wander, electrode motion, and muscle artifacts. The denoising results are then compared with those of state-of-the-art methods. Additionally, since no clean reference signal is available, the effectiveness of the proposed method is verified through visual comparison of its denoising results with those of other techniques on measured heartbeat signals. Finally, the advantages and disadvantages of enhanced DWT are analyzed and summarized.

## 2. Principle

### 2.1. ACF Evaluation Indicator

**Figure 1:**
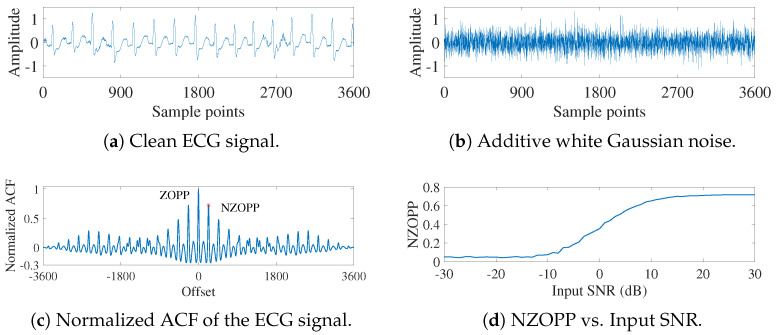
Normalized ACF of noisy ECG signals change with input SNR.

Our proposed metric, the nonzero-order periodic peak (NZOPP) of the normalized autocorrelation function (ACF) [36,59], has been successfully used to evaluate the quality of denoised signals and to obtain optimal filter parameters. It is defined as

(1) $$r_{xx}\left(m\right)=\frac{\sum_{n=0}^{N-1-|m|}\left(x\left(n\right)-\bar{x}\right)\left(x\left(n+m\right)-\bar{x}\right)}{\sum_{n=0}^{N-1}{\left(x\left(n\right)-\bar{x}\right)}^{2}},$$

where x(n) is the clean signal, $\bar{x}$ represents the mean value of signal x(n) , *N* denotes the signal sequence length, *n* is the signal sequence number, and *m* is the delay. This metric leverages the fundamental principle that signals exhibit autocorrelation while AWG noise does not, as illustrated in Figure 1.

Adding 5 dB AWG noise (Figure 1b) to a clean ECG signal (Figure 1a) creates a noisy signal whose NZOPP is shown by the red asterisk in Figure 1d. As the AWG noise varies from −30 to 30 dB, the NZOPP increases monotonically with the SNR, as shown in Figure 1d. This demonstrates that the NZOPP can serve as an alternative to SNR for evaluating the quality of denoised signals without requiring knowledge of the clean signals [37].

(2) $$\mathrm{SNR}={10\log}_{10}\left\{\sum_{n=1}^{N}\frac{x{\left(n\right)}^{2}}{{\left[x(n)-y(n)\right]}^{2}}\right\},$$

(3) $$\mathrm{RMSE}=\sqrt{\frac{1}{N}\left(\sum_{n=1}^{N}{\left[x(n)-y\left(n\right)\right]}^{2}\right)},$$

In addition, there is a measure of the difference between the clean and denoised signals, i.e., the root mean square difference percentage (PRD) [60].

(4) $$\mathrm{PRD}=\sqrt{\sum_{n=1}^{N}\frac{{\left[x(n)-y(n)\right]}^{2}}{x{\left(n\right)}^{2}}}\times 100\%$$

The metric to analyze the distortion level of the signal before and after denoising, Signal-to-Noise and Distortion Ratio (SINAD), can be expressed as

(5) $$\mathrm{SINAD}=10\times \log_{10}\left(\frac{S}{N+D}\right)$$

where *S* stands for the signal power, *N* for the noise power, and *D* for the nonlinear distortion power. Thus, the larger the SINAD, the smaller the distortion degree.

### 2.2. Improved Threshold and Threshold Function

**Figure 2:**
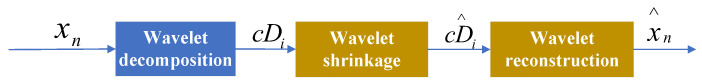
Wavelet-Based denoising process.

The wavelet transform is a time-frequency analysis tool that can be used for signal processing and denoising. The principle of wavelet transform is shown in Figure 2. Specific mathematical background and implementation details can be found in reference [61].

Wavelet denoising primarily involves four key parameters: the wavelet basis, decomposition level, threshold, and threshold function. The choice of mother wavelet, which serves as the basis waveform for generating wavelet functions, is critical to the performance of the denoising process. Commonly used mother wavelets include Haar, Daubechies (db), Symlets, and Coiflets. Among these, Daubechies wavelets are particularly well-suited for noise reduction in ECG signals [62,63]. Their excellent time-frequency localization capabilities enable the effective capture of rapid changes and subtle features inherent in ECG signals. Additionally, higher-order Daubechies wavelets preserve signal details through an increased number of vanishing moments, making them highly effective for suppressing low-frequency noise. Like other wavelet families (e.g., Haar, Symlets, and Coiflets), db wavelets possess orthogonality and perfect reconstruction (PR) properties, which ensure that critical information within the ECG signal is retained during both decomposition and reconstruction. Moreover, the availability of different orders offers flexibility in optimizing noise reduction according to the specific characteristics of the signal. In contrast, Haar wavelets, which are equivalent to db1, are computationally simple but have limited vanishing moments and frequency resolution. This can result in insufficient detail capture for complex ECG signal features. Symlet wavelets offer improved symmetry compared to Daubechies wavelets, but their lower-order variants may struggle to adequately address high-frequency noise and transient features. Coiflet wavelets exhibit greater symmetry and asymptotic properties than Daubechies wavelets, but their limited higher-order characteristics can reduce their effectiveness for processing complex and rapidly changing transient signals. For these reasons, Daubechies wavelets are widely preferred for ECG signal processing. While higher-order Daubechies wavelets generally provide improved vanishing moments and better time-frequency localization, leading to enhanced frequency resolution and signal feature capture, we balanced processing efficiency with the accuracy of signal processing. After careful consideration, we selected the db3 wavelet basis for our study, as it offers a suitable compromise between computational speed and denoising performance. To determine the optimal decomposition level, we decomposed the ECG signal into eight layers of wavelet coefficients. These coefficients were then processed layer by layer using wavelet thresholding and threshold functions, while the SNR and RMSE of the reconstructed signal were evaluated to assess the denoising performance. A typical wavelet generalized threshold is

(6) $$\lambda =\sigma \sqrt{2\log N},$$

where λ represents the wavelet threshold, and σ is the noise standard deviation. Unfortunately, noise standard deviation estimation is challenging in practical scenarios. Subsequently, the wavelet coefficients containing the noise coefficients need to be shrunk using a threshold function to remove the noise. Soft and hard thresholding functions are commonly used, which are expressed as Equations (7) and (8), respectively:

(7) $$\hat{\omega }=\left\{\begin{array}{cc} \omega , & |\omega_{j,k}|\geq \lambda ,\\ 0, & |\omega |<\lambda , \end{array}\right.$$

(8) $$\hat{\omega }=\left\{\begin{array}{cc} \mathrm{sgn}(\omega )*(\omega -\lambda ), & |\omega |\geq \lambda ,\\ 0, & |\omega |<\lambda , \end{array}\right.$$

where $\hat{\omega }$ and ω represent the wavelet coefficients before and after the threshold processing, respectively, sgn(*) is the sign function. Hard thresholding methods outperform soft thresholding methods in terms of mean square error, but they may introduce additional oscillations and jump points in the signal, thereby reducing signal smoothness. In contrast, soft thresholding maintains the overall continuity of the wavelet coefficients and reduces additional oscillations. However, it tends to compress the signal and introduce a certain bias, which directly affects the approximation of the reconstructed signal to the true signal. To mitigate the pseudo-Gibbs phenomenon and the constant bias issues associated with both soft and hard thresholding functions, Andrew Bruce and Hong-Ye Gao proposed a semi-soft threshold function, and the formula is shown as follows, $\lambda_{1}$ and $\lambda_{2}$ are low and high thresholds, respectively, refs. [64,65].

(9) $$\hat{\omega }=\left\{\begin{array}{cc} 0, & \mathrm{if}\;|\omega |\leq \lambda_{1}\\ \mathrm{sgn}\left(\omega \right)\frac{\lambda_{2}\left(|\omega \right|-\lambda_{1})}{\lambda_{2}-\lambda_{1}}, & \mathrm{if}\;\lambda_{1}<\left|\omega \right|\leq \lambda_{2}\\ \omega , & \mathrm{if}\;|\omega |>\lambda_{2} \end{array}\right.$$

Based on the continuity of the semi-soft threshold function, we propose an improved threshold function based on the hyperbolic tangent function,

(10) $$\hat{\omega }=\mathrm{sgn}\left(\omega \right)*\frac{\left|\omega \right|}{2}*\left\{\tanh\left[\alpha *\left(\left|\omega \right|-\lambda \right)\right]+1\right\}.$$

Simplifying Equation (10), we obtain

(11) $$\hat{\omega }=\mathrm{sgn}\left(\omega \right)*\left|\omega \right|*\left\{\frac{\exp[2\alpha (|\omega |-\lambda )]}{1+\exp[2\alpha (|\omega |-\lambda )]}\right\}.$$

The asymptote of Equations (10) and (11) as α tends to infinity is y=x , i.e., it approximates the hard threshold function. The proof procedure is shown in Equations (12) and (13). Let x=ω , when x>0 ,

(12) $$\begin{array}{cc} \; & \begin{array}{cc} \; & \lim_{x\to +\infty }\frac{\mathrm{sgn}\left(x\right)\cdot \left|x\right|\cdot \left[\tanh[\alpha \cdot (|x|-\lambda )]+1\right]}{2x}\\ \; & \;=\frac{-\left|x\right|\cdot \left[\tanh(+\infty )+1\right]}{2x}=1. \end{array} \end{array}$$

Similarly, when x<0 ,

(13) $$\begin{array}{cc} \; & \begin{array}{cc} \; & \lim_{x\to -\infty }\frac{\mathrm{sgn}\left(x\right)\cdot \left|x\right|\cdot \left[\tanh[\alpha \cdot (|x|-\lambda )]+1\right]}{2x}\\ \; & \;=\frac{-\left|x\right|\cdot \left[\tanh(+\infty )+1\right]}{2x}=1. \end{array} \end{array}$$

In this study, Gaussian white noise was added to a clean square wave signal, which was then denoised using four different threshold functions: the soft threshold function, the hard threshold function, the semi-soft threshold function, and the improved threshold function proposed herein. The resulting output SNRs are presented in Table 1. Notably, the input SNR for the semi-soft threshold function is higher than that of both the soft and hard threshold functions. Moreover, the improved threshold function demonstrates superior performance across a range of noise intensities, from −5 dB to 25 dB. The maximum output SNR is achieved within this noise intensity range, with the improved threshold function outperforming traditional threshold functions.

**Figure 3:**
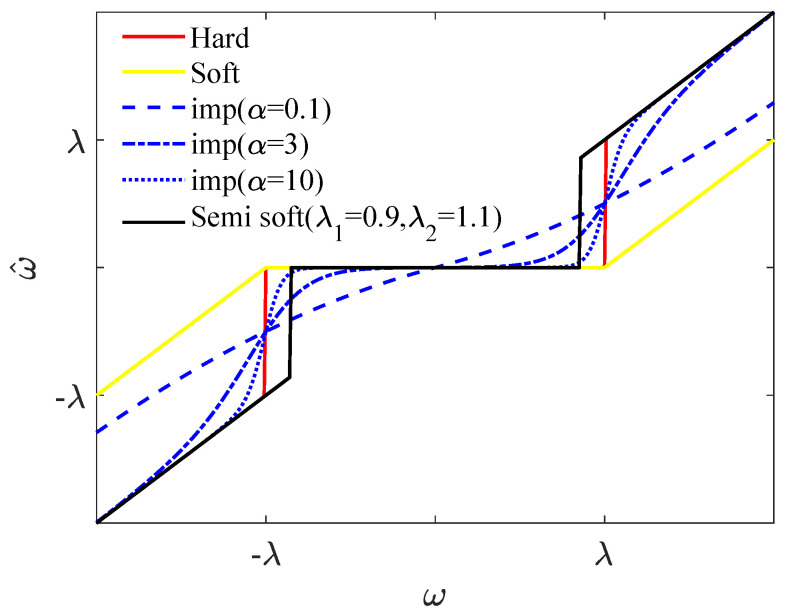
Comparison of the proposed threshold function with the soft, semi-soft and hard threshold functions.

The semi-soft thresholding function, represented by the black solid line in Figure 3, achieves a smooth transition between soft and hard thresholding by adjusting the upper and lower thresholds. However, this method is constructed based on a piecewise function, which lacks high-order differentiability. Regarding the improved function, when α = 10, the proposed threshold function is represented by the dash-dot line in Figure 3, which is closer to the hard threshold function than the curve (dotted line) corresponding to α = 3. As α approaches zero ( α = 0.1), the proposed threshold function curve becomes more similar to the soft threshold in the region where λ is significantly higher, while remaining closer to the hard threshold function in regions away from λ , as illustrated by the dashed line. It is well established that the soft threshold function was introduced to mitigate the pseudo-Gibbs phenomenon associated with the hard threshold function; however, it introduces a constant deviation. In contrast, the proposed threshold function does not approximate the soft threshold as α approaches zero. Instead, it effectively avoids the constant deviation characteristic of the soft threshold while preserving the continuity of the function around λ . Additionally, the improved wavelet threshold function employs a non-segmented basis function, which is continuously differentiable at higher orders. This approach addresses the issue of discontinuity in the higher-order derivatives of traditional threshold functions and alleviates the signal oscillations caused by hard thresholding. Furthermore, the function can be adjusted using the factor alpha to minimize the constant deviation problem. However, selecting the optimal wavelet parameters, particularly the wavelet threshold and threshold function, remains a challenge. The next subsection of this paper discusses the use of an objective evaluation metric to determine the optimal wavelet threshold and tuning factor.

α is a tuning factor used to adjust the speed at which the improved threshold function approaches the hard threshold function continuously at the threshold λ . The comparison between the improved threshold function and the soft and hard threshold functions is shown in Figure 3. When α=0.1 , the improved threshold function approaches the hard threshold function slowly in the interval of |ω|≫λ with ω≠0 . When α=10 , the improved threshold function quickly approaches the hard threshold function. Therefore, different α enable the improved threshold function to control the speed approaching the hard threshold function. If α is appropriate, the improved threshold function has the best denoising effect.

### 2.3. Fast Algorithms for Thresholds and Threshold Function

**Figure 4:**
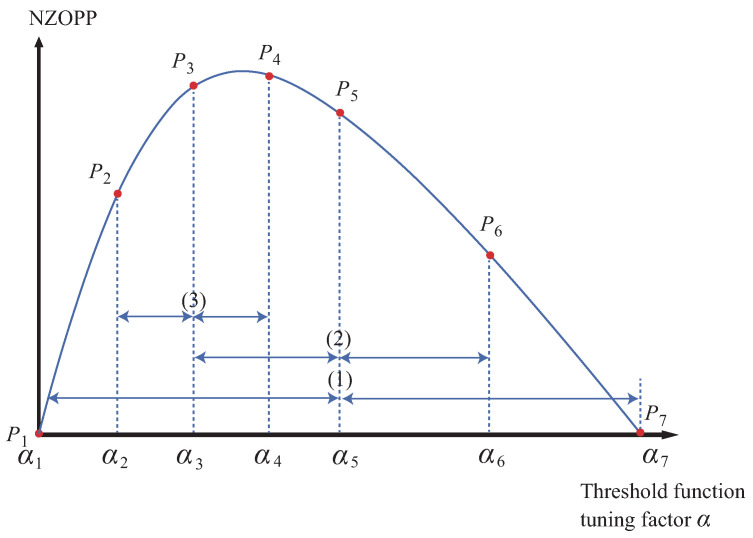
Schematic of fast bisection interpolation method for wavelet threshold function.

To efficiently determine the threshold *T* and the tuning factor α , we have proposed a bisection fast algorithm based on the evaluation metric (NZOPP) [36], as shown in Figure 4.

According to the definition of SNR, a maximum SNR exists as the wavelet threshold changes from a small value to a large value. When the wavelet threshold is optimized, the NZOPP of the denoised signal, serving as an alternative measure of the SNR, reaches its maximum value [36,37]. The basic idea of Figure 4 is to keep conducting bisection interpolation within the preset wavelet threshold lookup range until the NZOPP reaches the maximum. The steps to find the exact wavelet threshold *T* have been described in detail in Ref. [37]. The focus of this paper describes the steps to find the optimal tuning factor α of the threshold function.

(1) Set the wavelet basis as db3, perform an 8-layer wavelet decomposition, and set a sufficiently wide tuning factor α in the range [ $\alpha_{0},\alpha_{m}$ ] with the interpolation $\alpha_{i}=\left(\alpha_{0}+\alpha_{m}\right)/2$ , i.e., [ $\alpha_{1},\alpha_{5},\alpha_{7}$ ], as shown in the step (1) of Figure 4;
(2) Denoise the noisy signals with each tuning factor of threshold function in [ $\alpha_{1},\alpha_{5},\alpha_{7}$ ] and calculate the NZOPP corresponding to each denoised signal to obtain [ $P_{0},P_{i},P_{m}$ ], i.e., [ $P_{1},P_{5},P_{7}$ ];
(3) Find the maximum NZOPP. For example, $P_{5}$ and its corresponding $\alpha_{5}$ ;
(4) Interpolate both sides of $\alpha_{5}$ to generate a new sequence [ $\alpha_{1},\left(\alpha_{1}+\alpha_{5}\right)/2,\alpha_{5}$ , $\left(\alpha_{5}+\alpha_{7}\right)/2,\alpha_{7}$ ], i.e., [ $\alpha_{1},\alpha_{3},\alpha_{5},\alpha_{6},\alpha_{7}$ ], as shown in the step (2) of Figure 4.
(5) Denoise the noisy signals with each new tuning factor of threshold function ( $\alpha_{3},\alpha_{6}$ ) and calculate the NZOPP corresponding to each denoised signal to obtain [ $P_{1},P_{3},P_{5},P_{6},P_{7}$ ];
(6) Find the maximum value in [ $P_{1},P_{3},P_{5},P_{6},P_{7}$ ]. For example, $P_{3}$ and its corresponding $\alpha_{3}$ ;
(7) Interpolate on both sides of $\alpha_{3}$ to obtain a new tuning factor sequence [ $\alpha_{1}$ , $\left(\alpha_{1}+\alpha_{3}\right)/2,\alpha_{3},\left(\alpha_{3}+\alpha_{5}\right)/2,\alpha_{5},\alpha_{6},\alpha_{7}$ ], i.e., [ $\alpha_{1},\alpha_{2},\alpha_{3},\alpha_{4},\alpha_{5},\alpha_{6},\alpha_{7}$ ], as shown in the step (3) of Figure 4;
(8) Repeat steps (4)∼(7) until $\alpha_{i}-\alpha_{i+1}<10^{-10}$ ;
(9) Outputs the current $\alpha_{i}$ as the optimal tuning factor.

## 3. Simulation Experiments

To validate the effectiveness of the enhanced DWT, we conducted denoising experiments comparing its performance with traditional DWT and the state-of-the-art denoising methods on various electrocardiogram (ECG) signals contaminated with different types of noise. Then, the verified enhanced DWT was applied to denoise the real heartbeat signal measured by the fiber optic sensing mattress. The ECG signals were obtained from the MIT-BIH arrhythmia database (data No. 106, 109, 118, 210 and 233), Clayton University ventricular arrhythmia database (No. cu03, cu07, cu11, cu18 and cu30), and PTB diagnostic ECG database (No. s0016lrem, s00261rem, s0031lrem, s0038lrem, s0057lrem). The MIT-BIH arrhythmia database contains 30-min, two-channel ECG recordings sampled at 360 Hz, covering a wide range of arrhythmias such as atrial fibrillation and premature ventricular contractions. The Clayton University ventricular arrhythmia database focuses on severe ventricular arrhythmias (e.g., ventricular tachycardia and fibrillation) with recordings sampled at 250 Hz. The PTB diagnostic ECG database provides high-resolution, 15-lead ECG signals sampled at 1 kHz, including both healthy subjects and patients with cardiac conditions such as myocardial infarction and heart failure. The MIT-BIH noise stress test database provides real noise signals, including baseline wandering, electrode motion artifact, and muscle artifact. Baseline wandering, a low-frequency noise typically below 0.5 Hz, arises from respiratory movements or poor electrode–skin contact, causing slow drifts in the signal baseline. Electrode motion artifact, with a frequency range of 0.5 Hz to 10 Hz, results from relative motion between the electrode and the skin, leading to irregular fluctuations and sudden spikes. Muscle artifact, a high-frequency noise ranging from 20 Hz to 500 Hz, is generated by muscle activities, such as patient movement or tremors, manifesting as rapid oscillations that obscure high-frequency ECG features.

The AWG noise was added to the clean ECG signals using MATLAB’s awgn function, which specifies the signal-to-noise ratio (SNR) to control the noise intensity. The SNR values were set in the range of −10 dB to 30 dB with increments of 5 dB to simulate different noise levels. The noise was generated and superimposed on the clean signals to create contaminated ECG data for evaluating the denoising performance. The above-mentioned tests were conducted using MATLAB R2022b software on a 64-bit Windows 11 operating system.The computer used for the test was equipped with a 2.70 GHz Intel Core i7 processor (Intel Corporation, Santa Clara, CA, USA), 16 GB of RAM, and an NVIDIA GeForce RTX 3060 GPU (NVIDIA Corporation, Santa Clara, CA, USA) for accelerated computation. The Signal Processing Toolbox and Wavelet Toolbox were utilized to implement the denoising algorithms, ensuring compatibility and efficiency. The experimental workflow includes the following steps: (1) loading the clean ECG signals and noise data, (2) adding noise to the signals at specified SNR levels, (3) applying the traditional DWT, enhanced DWT, and state-of-the-art denoising methods, and (4) evaluating the performance using metrics, such as SNR, RMSE, and visual inspection.

### 3.1. ECG Signals Contaminated by AGW Noise

**Figure 5:**
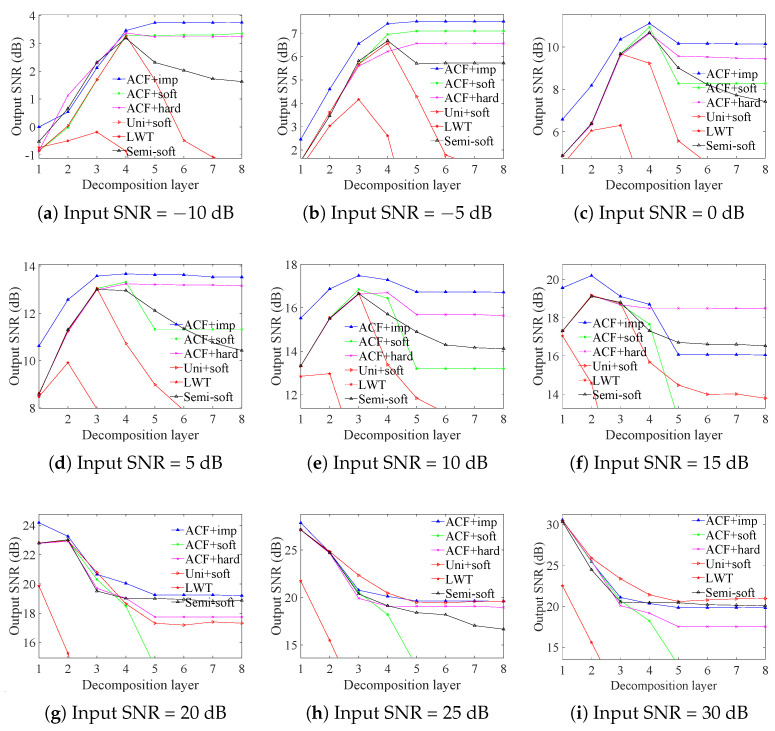
Comparison of improved wavelet thresholding and thresholding functions for denoising ECG signals.

We initially employed the enhanced discrete wavelet transform (DWT) to denoise the ECG signal recordings of No. 109 contaminated by additive white Gaussian (AWG) noise and compared the results with those obtained from other methods, as illustrated in Figure 5. Subsequently, the effectiveness of the proposed method was validated using five additional types of ECG signals, each contaminated by AWG noise. After achieving the precise threshold, the output SNRs of enhanced DWT at the optimal decomposition layer were sightly higher than those of traditional methods. Specifically, the output SNR of the denoised signal obtained by the improved threshold and soft threshold function (green line) is greater than that obtained by the Universal threshold and soft threshold function (red lines). Furthermore, by enhancing the threshold function, the results were further improved: the output SNR of the denoised signal obtained by the improved threshold and the improved function (blue line) is greater than that obtained by the improved threshold and soft/hard threshold function (green/magenta lines), and the output SNR obtained by enhanced DWT at different noise levels is greater than that of the semi-soft threshold function (black line). This enhancement effectively mitigated the pseudo Gibbs phenomenon and constant deviation. To ensure robustness against the randomness of the noise distribution, we averaged the output SNR and RMSE of the optimal decomposition layer obtained by the enhanced and traditional DWT, along with the state-of-the-art methods (EMD + DWT, VMD + DWT, EEMD + LM), over 100 iterations. The results clearly demonstrate that the enhanced DWT achieved the highest output SNR (highlighted in bold in the first column of Table S1 in the Supplementary Material S1) and the lowest RMSE (highlighted in bold in the first column of Table S2 in the Supplementary Material S1). Thus, the enhanced DWT significantly outperforms traditional DWT and other denoising methods across input SNR levels ranging from −10 to 30 dB. When the input signal-to-noise ratio is low, the autocorrelation of finite-length signals cannot completely suppress the noise causing NZOPP fluctuations. For example, when the input SNR is −10 dB, the finite-length large magnitude noise sequence is not completely uncorrelated. As a result, the NZOPP metrics show non-monotonic variations due to this effect. Above −10 dB input SNR, the NZOPP metric is more stable and directly reflects the signal quality. The Enhanced DWT uses the NZOPP metric to find the exact threshold and the optimal threshold function. Therefore, the wavelet coefficients representing signal and noise components can be accurately differentiated, and then the wavelet coefficients can be further processed by the adaptive threshold function. The above experiments show that the method can retain the most complete signal components while removing as much noise as possible, i.e., the RMSE is minimized and the SNRimp is maximized.

**Figure 6:**
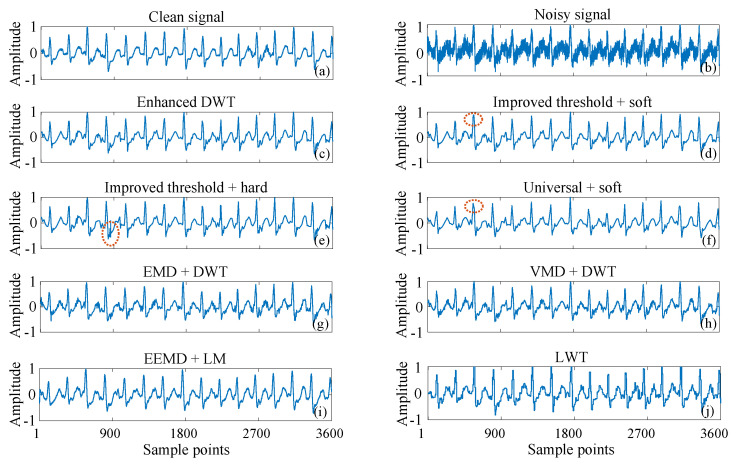
Visual comparison of the denoising waveforms of the 109 ECG signal with 5 db AWG noise (**a**) Clean signal, (**b**) Noisy signal, (**c**) Enhanced DWT (improved threshold + improved threshold function), (**d**) Improved threshold + soft, (**e**) Improved threshold + hard, (**f**) Universal + soft, (**g**) EMD + DWT, (**h**) VMD + DWT, (**i**) EEMD + LM, (**j**) LWT.

To visualize the denoising efficacy of the proposed method, Figure 6 compares denoising results across various methods for 109 ECG signals contaminated with AWG noise. Figure 6a–c shows the clean ECG signal, the noise-containing signal with 5 dB AWG noise, and the denoised signal using the enhanced DWT method, respectively. The denoising results indicate that the useful signal in Figure 6c is more accurately preserved compared to the signal highlighted by the red dashed circle in Figure 6d using the Improved Threshold + Soft method. Specifically, Figure 6c demonstrates reduced oscillations at the edges of the P-peak compared to the signal highlighted by the red dashed circle in Figure 6e using the Improved Threshold + Hard method. This demonstrates that the improved threshold function not only minimizes the constant deviation associated with the soft threshold method but also effectively mitigates the pseudo-Gibbs phenomenon observed with the hard threshold method. Furthermore, compared to the conventional DWT method (Figure 6f), the denoising curve produced by the Improved Threshold + soft method (Figure 6d) shows less distortion around the red dashed circle. This indicates that our proposed improved thresholding method achieves more accurate denoising results. Meanwhile, the noise level of the denoised ECG signals using the enhanced DWT is significantly lower than those of the state-of-the-art techniques, including EMD + DWT, VMD + DWT, and EEMD + LM, LWT. Therefore, the enhanced DWT method surpasses traditional DWT techniques and even outperforms existing composite denoising methods.

To assess the accuracy and real-time performance of various methods for denoising heartbeat shock signals, we compared the output SNR and computational time required by six different denoising methods applied to ECG signals contaminated with 5 dB of AWG noise. The proposed algorithm needs to try dichotomous interpolation in determining the optimal threshold and the tuning factor of the threshold function and to evaluate the quality of the denoised ECG signal using these values. Therefore, finding the appropriate interpolation values is the most time-consuming. To improve the computational efficiency, we do parallel ECG signal denoising for the two inserted tuning factor values. As shown in Table 2, the enhanced DWT technique achieves the highest output SNR of 14.4542 dB, accomplishing this in a mere 0.2597 s. This elapsed time is more than that of the conventional wavelet method but is less than those of those composite methods. If further improvement of the algorithm efficiency is needed, a parallel computational method with three or four times interpolation can also be used.

**Figure 7:**
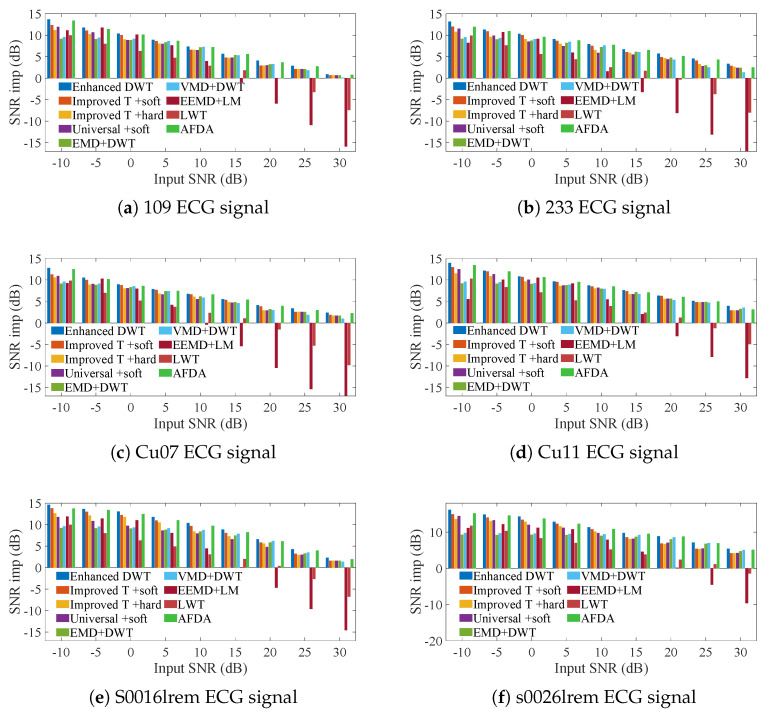
Comparison of the SNRs of the enhanced DWT and the combination methods for denoising 6 ECG signals containing different AWG noise levels.

To demonstrate the generality of the proposed method under different signal and noise distributions, we compared the denoising results of various state-of-the-art denoising methods for ECG signals from different databases, as shown in Figure 7.

To mitigate the influence of AWG noise distribution, the output SNR of denoised signals with identical input noise levels was averaged across 100 iterations. The enhanced DWT (Improved Threshold + Improved Threshold Function) consistently exhibits significantly higher output SNR compared to the improved DWG (Improved Threshold + soft/hard threshold function) and notably outperforms the conventional DWT (Universal + soft threshold function) across ECG denoised signals ranging from input SNRs of −10 dB to 30 dB. Importantly, it even surpasses composite denoising methods, such as EMD + DWT, VMD + DWT, EEMD + LM, and Activation Function Dynamic Averaging (AFDA). Specific SNR and RMSE are detailed in Tables S1 and S2 of the Supplementary Material S1. The superiority of the enhanced DWT method can be attributed to objective evaluation metrics that accurately determine wavelet thresholds, coupled with an improved wavelet threshold function that effectively mitigates pseudo-Gibbs oscillations and constant deviations.

**Figure 8:**
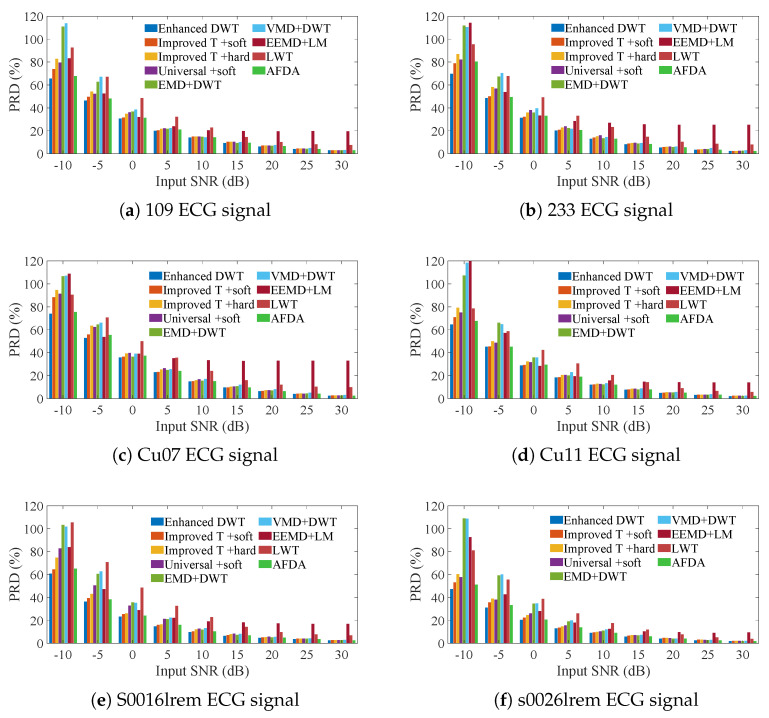
Comparison of the SNRs of the enhanced DWT and the combination methods for denoising 6 ECG signals containing different AWG noise levels.

To ensure robustness to the randomness of the noise distribution, we averaged the PRDs of the best decomposition layers obtained in 100 iterations for the enhanced DWT and the conventional DWT, as well as the state-of-the-art methods (EMD + DWT, VMD + DWT, EEMD + LM, LWT). The results show that the enhanced DWT achieves the smallest PRD in the input SNR range of −10 to 30 dB as shown in Figure 8 (see Table S9 in Supplementary Material S1 for detailed data). That is, the enhanced DWT changes the original signal slightly less than the AFDA and significantly less than the conventional DWT and the state-of-the-art composite denoising methods, such as EMD + DWT, VMD + DWT, and EEMD + LM. This is attributed to the fact that the objective evaluation metric (NZOPP) can accurately determine the wavelet threshold and the tuning factor α of the threshold function.

**Figure 9:**
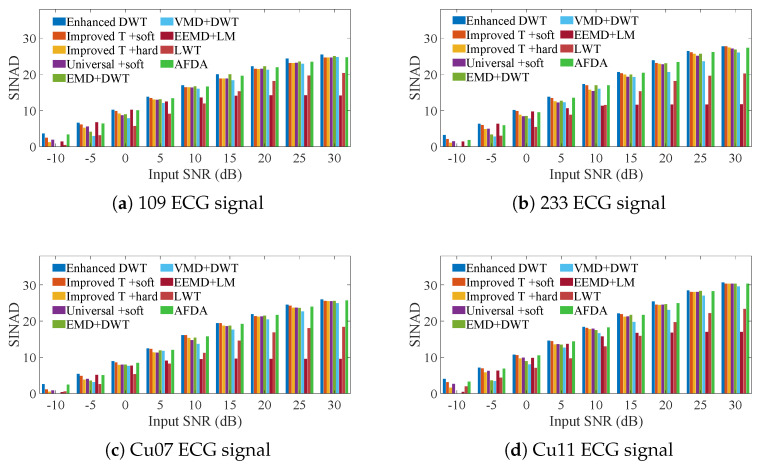

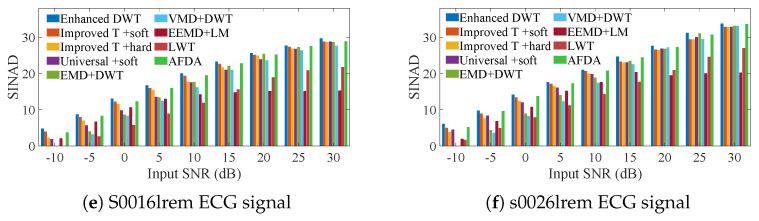
Comparison of the SNRs of the enhanced DWT and the combination methods for denoising 6 ECG signals containing different AWG noise levels.

To evaluate the distortion of the denoised signals and assess the fidelity of the proposed method, we introduced Gaussian white noise at various input SNRs to different ECG signals and calculated the SINAD of the denoised signals. The results, presented in Figure 9 (specific data can be found in Supplementary Material S1), indicate that the fidelity of the denoised signals obtained through the enhanced DWT method surpasses that achieved by traditional wavelet methods combined with other techniques, including EMD + DWT, VMD + DWT, EEMD + LM and AFDA.

### 3.2. ECG Signals Contaminated by Real Noise

**Figure 10:**
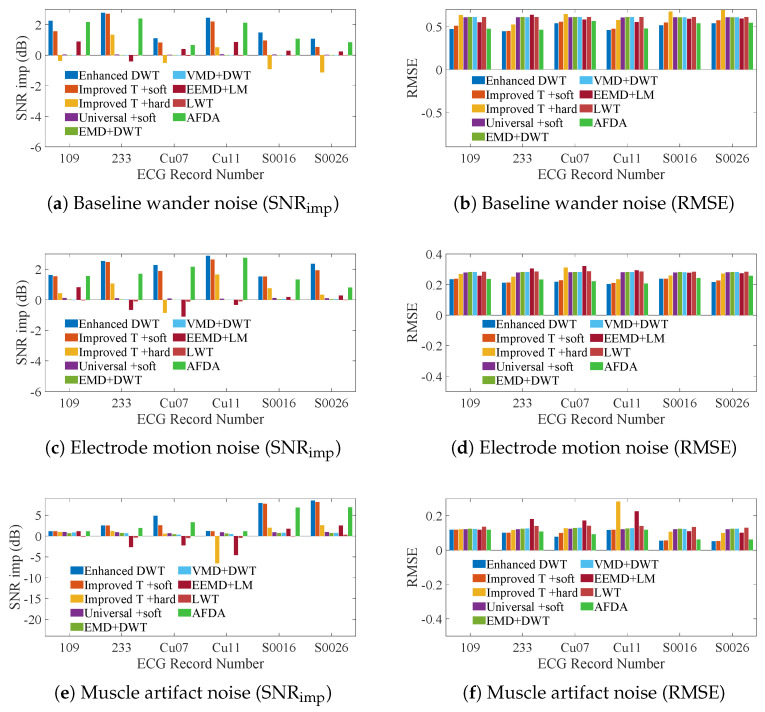
Comparison of the SNRs of the enhanced DWT and the combination methods for denoising 6 ECG signals containing different AWG noise levels, (**a**) 109 ECG signal, (**b**) 233 ECG signal, (**c**) cu07 ECG signal, (**d**) cu11 ECG signal, (**e**) s0016lrem ECG signal, and (**f**) s0026lrem ECG signal.

To assess the practicality of enhanced DWT, we conducted denoising experiments on ECG signals contaminated with practical noises, such as baseline wander noise, electrode motion noise, and muscle artifact noise. We compared the SNR gain ${\mathrm{(SNR}}_{\mathrm{imp}}$ = ${\mathrm{SNR}}_{\mathrm{out}}$ − ${\mathrm{SNR}}_{\mathrm{in}}$ ) and RMSE of various denoising methods for noise reduction of noisy ECG signals, as shown in Figure 10.

A comparison of the SNR gains and RMSE obtained by different noise reduction methods for ECG signals contaminated by baseline wander noise is shown in Figure 10a and Figure 10b, respectively, (shown in Tables S3 and S4 of the Supplementary Material S1, respectively). The ${\mathrm{SNR}}_{\mathrm{imp}}$ obtained by the enhanced DWT surpassed that of the existing method and even the AFDA method, while the RMSE was significantly lower. It is worth noting that the Improved threshold + soft method obtained a higher ${\mathrm{SNR}}_{\mathrm{imp}}$ than the other methods, while the enhanced DWT (Improved threshold + Improved threshold function) further improved ${\mathrm{SNR}}_{\mathrm{imp}}$ .

A comparison of the SNR gains and RMSE obtained by different noise reduction methods for ECG signals contaminated by electrode motion noise is shown in Figure 10c and Figure 10d, respectively, (shown in Tables S5 and S6 of the Supplementary Material S1, respectively). The enhanced DWT exhibited superior performance with a much larger ${\mathrm{SNR}}_{\mathrm{imp}}$ and smaller RMSE compared to existing methods. The enhanced DWT with improved threshold function outperformed all other techniques.

A comparison of the SNR gains and RMSE obtained by different noise reduction methods for ECG signals contaminated by the muscle artifact noise is shown in Figure 10e and Figure 10f, respectively, (shown in Tables S7 and S8 of the Supplementary Material S1, respectively). The enhanced DWT demonstrated significantly higher ${\mathrm{SNR}}_{\mathrm{imp}}$ and lower RMSE than the other methods. Particularly, the enhanced DWT with the improved threshold and soft threshold function already outperformed other methods and occasionally underperformed the AFDA method. The improved thresholding function further improves the DWT denoising capability and makes it superior to existing methods.

Baseline wander noise belongs to low-frequency noise, and its frequency is usually lower than 0.15 Hz; electrode motion noise usually exhibits sudden high-frequency noise with a wide frequency range (0.1 Hz 10 Hz); muscle artifacts are high-frequency noise with a frequency range of 5 Hz–100 Hz. Since the noise-containing signals are mainly denoised for high-frequency noise during the decomposition process, enhanced DWT performs more significantly in removing the electrode motion noise and muscle artefact noise and is able to achieve higher output SNR and smaller RMSE. In addition, since the method proposed in this paper is based on the correlation between the signals themselves, enhanced DWT does not need to pre-assume the distributional characteristics of the noise, thus outperforming the other comparative methods in terms of SNR increment and RMSE metrics.

**Figure 11:**
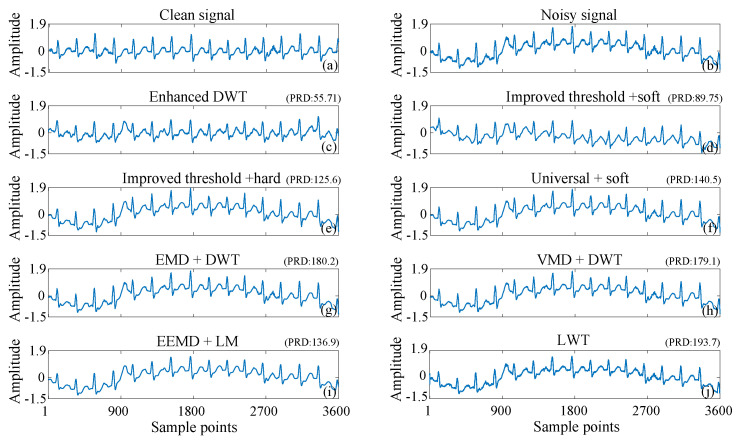
Visual comparison of the denoising results of the 109 ECG signal with baseline wander (**a**) clean signal, (**b**) noisy signal, (**c**) enhanced DWT, (**d**) improved threshold + soft, (**e**) improved threshold + hard, (**f**) universal + soft, (**g**) EMD + DWT, (**h**) VMD + DWT, (**i**) EEMD + LM, (**j**) LWT.

Baseline wander is a prevalent form of noise in electrocardiogram (ECG) signals. It primarily arises from factors, such as respiration, variations in electrode–skin contact, and patient movement. The defining characteristic of baseline wander is its slow variation over an extended time scale, resulting in a vertical displacement of the entire ECG signal. Baseline wander adversely affects the morphology of the ECG signal, complicating accurate measurement and analysis of the waveform. Consequently, it may lead to the misinterpretation of critical points within the ECG signal, including the identification of the QRS complex. For visualization, Figure 11 illustrates denoising outcomes from various methods applied to 109 ECG signals contaminated by baseline wander noise. Figure 11a,b depicts clean ECG signals and those contaminated by baseline wander noise, respectively. Figure 11c displays the denoising results using enhanced DWT, while Figure 11d–j showcases the results from other existing methods. It is evident that enhanced DWT effectively removes baseline wander noise, demonstrating significantly smaller fluctuations in signal amplitude compared to other noise reduction methods.

**Figure 12:**
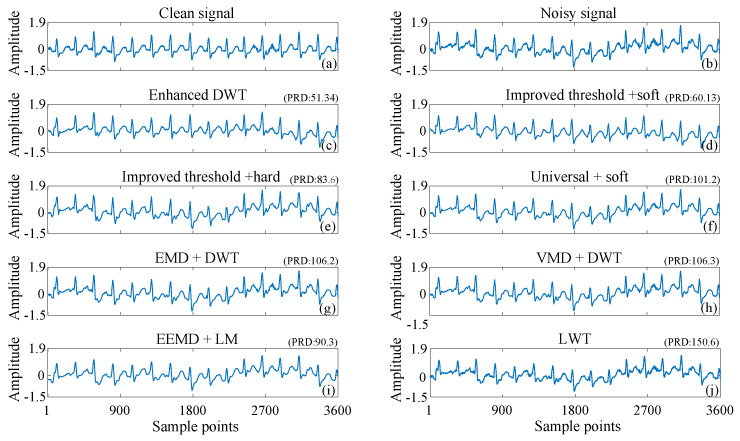
Visual comparison of the denoising results of the 109 ECG signal with electrode motion (**a**) Clean signal, (**b**) Noisy signal, (**c**) Enhanced DWT, (**d**) Improved threshold + soft, (**e**) Improved threshold + hard, (**f**) Universal + soft, (**g**) EMD + DWT, (**h**) VMD + DWT, (**i**) EEMD + LM, (**j**) LWT.

Electrode movement refers to alterations in the relative position of the electrode to the skin, which can induce additional irregularities in the ECG. Potential causes of this phenomenon include the patient’s physical activity, respiration, and muscle tension. Figure 12 compares the denoising outcomes of various methods applied to ECG signals contaminated by electrode motion noise. Figure 12a and Figure 12b presents a clean ECG signal and a noisy signal contaminated by electrode motion, respectively. Figure 12c displays the denoising results obtained using enhanced DWT, while Figure 12d–j showcases results from other existing methods. The comparison shows that enhanced DWT can effectively mitigate the morphological distortion caused by electrode motion noise on ECG. It preserves the signal components more completely and removes the noise to the maximum extent.

**Figure 13:**
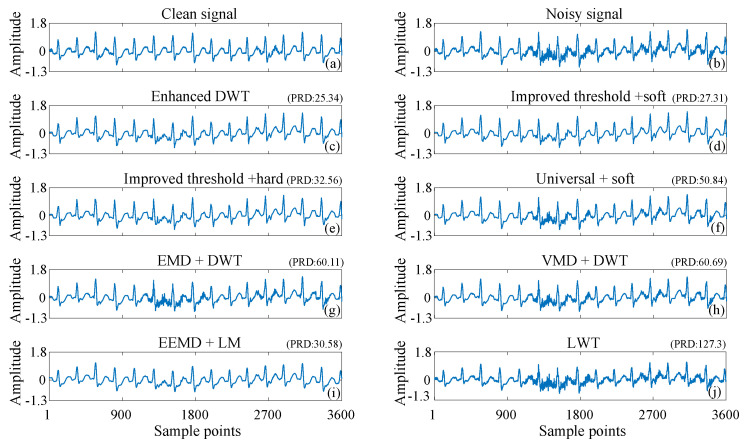
Visual comparison of the denoising results of the 109 ECG signal with muscle artifact (**a**) Clean signal, (**b**) Noisy signal, (**c**) Enhanced DWT, (**d**) Improved threshold + soft, (**e**) Improved threshold + hard, (**f**) Universal + soft, (**g**) EMD + DWT, (**h**) VMD + DWT, (**i**) EEMD + LM, (**j**) LWT.

Muscle artifacts are disturbances in the electrocardiogram (ECG) signal caused by random and irregular electrical activity generated during muscle contraction and relaxation. These artifacts typically exhibit a relatively wide frequency range, generally between 10 Hz and several hundred Hz. This broad frequency spectrum complicates the distinction of true ECG features and may result in erroneous diagnoses of heart disease. Figure 13 compares the denoising curves of different denoising methods for ECG signals contaminated by muscle artifacts. Figure 13a and Figure 13b shows the clean ECG signal and the noisy signal contaminated by muscle artifacts, respectively. A comparison with the denoising results of other methods in Figure 13d–j shows that the denoising curve of the enhanced DWT in Figure 13c is closest to the clean one. Therefore, the proposed method can accurately remove artifacts and better preserve the characteristics of useful signals.

As shown in Figure 11, Figure 12 and Figure 13, the enhanced DWT method has a smaller PRD than the other methods for denoising ECG signals interfered with by baseline drift, electrode movement, and muscle artifacts. This indicates that the deviation of the denoised signal from the original clean signal is minimized by the enhanced DWT.

In conclusion, the proposed enhanced DWT exhibited superior denoising capabilities across various practical noise scenarios, outperforming existing methods in terms of SNR improvement and RMSE. The enhanced DWT, especially when coupled with an improved threshold function, proved to be highly effective in mitigating practical noise interference in ECG signals.

### 3.3. Heartbeat Signals Detected by Non-Invasive Technology

**Figure 14:**
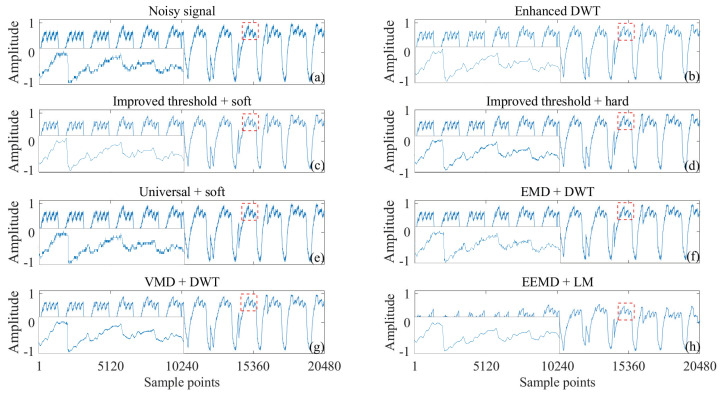
Visual comparison of the denoising results of the measured heartbeat signals (**a**) using different methods, including (**b**) enhanced DWT (improved threshold + improved threshold function), (**c**) improved threshold + soft, (**d**) improved threshold + hard, (**e**) universal + soft, (**f**) EMD + DWT, (**g**) VMD + DWT, (**h**) EEMD + LM.

The heartbeat signal is monitored using a 1 × 1 m² smart mattress equipped with a fiber optic sensor based on intermodal interference. A 650 nm laser excites higher-order modes in a 1 m multimode fiber (MMF), where physiological activities such as heartbeat and respiration alter the light intensity distribution. These variations are detected by a Thorlabs DET10A2 photodetector (Thorlabs Inc., Newton, NJ, USA), converted into electrical signals, amplified, digitized at 2048 Hz, and processed on a computer to extract heartbeat information. To validate the noise reduction effect of the enhanced DWT on the heartbeat signals for non-invasive detection, we conducted a visual comparison of the noise reduction results from various techniques applied to the fiber optic micro-vibration sensors, as shown in Figure 14. Since obtaining a clean signal with precise noise levels is challenging due to the nature of the measured heartbeat signal, a visual comparison was employed to assess the effectiveness of the noise reduction.

Figure 14b exhibits the clearest heartbeat profile with minimal noise interference between peaks compared to Figure 14c–j. This clarity can be attributed to the effectiveness of the enhanced DWT method in minimizing noise interference while preserving signal components as much as possible. In contrast, Figure 14h, while visually free of noise interference, shows a notable decrease in signal amplitude compared to Figure 14a. Notably, Figure 14c,d presents clearer results compared to Figure 14e, indicating that the improved wavelet threshold significantly enhances denoising quality. Moreover, Figure 14b demonstrates cleaner signal profiles compared to Figure 14c,d, highlighting the further improvement brought by the enhanced threshold function. A closer examination of the denoised waveforms (inset of Figure 14) reveals that the detailed waveforms obtained by enhanced DWT (Figure 14b) are comparable to those obtained by improved threshold + soft (Figure 14c), significantly outperforming other methods shown in Figure 14d–j. Thus, the enhanced DWT method surpasses alternative denoising techniques in effectively reducing noise and preserving the integrity of heartbeat signals. Section 3.1 and Section 3.2 have established the effectiveness and statistical validity of the proposed method for processing ECG signals from various databases contaminated with different types of noise. However, in practical applications, the unavailability of clean signals and the challenges associated with accurately estimating the noise level can lead to inaccuracies in the calculated values of SNR, RMSE, PRD, and SINAD. In this subsection, the effectiveness of the proposed method is validated using fiber optic heartbeat monitoring as a specific example of measured heartbeat signals.

## 4. Conclusions

To realize non-invasive real-time and accurate heartbeat detection for wearable devices, this paper proposes a wavelet denoising method based on the autocorrelation function evaluation metrics of the denoised signals with exact wavelet thresholding and adaptive thresholding function. The excellent denoising performance of the enhanced DWT is verified by comparing the denoising results with traditional wavelet methods and state-of-the-art techniques for many types of heartbeat signals, including Gaussian white noise, baseline drift, power line noise and muscle artifacts.

Denoising ECG signals contaminated with AWG noise, enhanced DWT improves the signal-to-noise ratio by 1–5 dB, along with a higher SINAD, and lower RMSE and PRD than conventional DWT and other state-of-the-art methods. These results show that the proposed method minimizes distortion while maximizing denoising.

In denoising ECG signals affected by real noise, the enhanced DWT obtains SNR and SINAD gains that are tens to hundreds of times higher than those obtained by other state-of-the-art techniques, while RMSE and PRD are substantially lower. This demonstrates that the proposed enhanced DWT can efficiently determine the wavelet threshold and threshold function even when the spectra of the signal and the noise are very similar.

Furthermore, when applied to heartbeat signals monitored by an actual fiber optic microvibration sensing system, the enhanced DWT yields visually clearer denoised waveforms compared to existing methods that lack clean signal references and accurate noise level estimations.

The enhanced DWT employs signal estimation rather than traditional noise level estimation to obtain wavelet thresholds and threshold functions. The autocorrelation function (ACF)-based wavelet threshold effectively distinguishes between spectrally similar signals and noise components, while the improved threshold function exhibits higher-order differentiability, significantly mitigating the pseudo-Gibbs phenomenon and reducing constant deviation. The proposed method uses the non-zero periodic peak of the autocorrelation function of a periodic signal as a noise-reduction quality assessment metric to select the appropriate wavelet threshold and threshold function. The method has no constraints except for the periodic signal. In addition, the effectiveness of this signal quality assessment index has been demonstrated by the non-local average noise reduction in φ -OTDR vibration signals [59], and it is applicable to the optimization of bilateral filtering parameters [36] and the threshold of DWT [37].

Limitations of the study: The proposed method is based on the non-zero periodic peak of the autocorrelation function (NZOPP), and therefore, it fails when dealing with non-periodic signals. For example, in the case of highly irregular heart rhythms (e.g., arrhythmias), the periodicity assumption no longer holds.

Future research challenges include the following three aspects: First, to address these limitations, future research on algorithms for processing nonperiodic signals, such as heartbeats with severe arrhythmia or other nonperiodic biosignals, is needed. Second, accurately separating and extracting heart rate and respiration frequency from complex physiological data based on noise reduction will be conducted; finally, embedding the proposed method into IoT devices (e.g., wearable sensors or smart medical systems) to enable continuous, real-time monitoring of physiological signals will be conducted (e.g., heartbeat and respiration patterns).

## Figures and Tables

**Table 1:** Comparison of output SNRs after denoising noisy signals with different threshold functions.

Input SNR (dB)	Soft	Hard	Semisoft	Improved
−10	9.8933	9.4434	10.4166	10.7973
−5	12.5791	12.7460	13.3488	13.6201
0	16.1215	15.8090	16.9137	17.5043
5	19.4142	19.6456	20.9539	21.7680
10	25.1601	25.1404	26.1945	26.8667
15	29.2865	29.2438	29.8761	30.4211

**Table 2:** Computational cost comparison.

Methods	Ehanced DWT	Improved T + Soft	Universal + Soft	EMD + DWT	VMD + DWT	EEMD + LM
Cost (s)	0.2597	0.1724	0.0960	0.3162	3.2624	0.4786
SNR (dB)	14.4542	13.9492	13.6104	13.7961	13.2239	12.6712

## Data Availability

Data are contained within the article.

## Supplementary Materials

The following supporting information can be downloaded at: https://www.mdpi.com/article/10.3390/s25061743/s1, Supplementary Material S1: Comparison of SNR, RMSE, PRD, SINAD between enhanced DWT and other methods for signal denoising.

## Abbreviations

The following abbreviations are used in this manuscript:

DWT	Discrete Wavelet Transform
AWG	Additive White Gaussian
AI	Artificial Intelligence
EMD	Empirical Mode Decomposition
VMD	Variable Mode Decomposition
SSA	Singular Spectrum Analysis
NLM	Non-Localized Mean Filtering
EEMD	Ensemble Empirical Mode Decomposition
CEEMD	Complementary Ensemble Empirical Mode Decomposition
IMF	Intrinsic Mode Functions
WT	Wavelet Transform
SDWT	Spcshrink’s Discrete Wavelet Transform
SWT	Stationary Wavelet Transform
SWPT	Stationary Wavelet Packet Transform
DTCWT	Dual Tree Complex Wavelet Transform
DDC	Double Density Complex
SBWT	Stationary Bionic Wavelet Transform
SNR	Signal-to-Noise Ratio
RMSE	Root Mean Square Error
MMF	Multimode Fiber
SMF	Single Mode Fiber
NZOPP	Nonzero-Order Periodic Peak
ACF	Autocorrelation Function
PRD	Root Mean Square Difference Percentage
SINAD	Signal-to-Noise and Distortion Ratio
ECG	Electrocardiogram
