# Correction: Zhu et al. Enhanced DWT for Denoising Heartbeat Signal in Non-Invasive Detection. *Sensors* 2025, *25*, 1743

**DOI:** 10.3390/s25103050

**Published:** 2025-05-12

**Authors:** Peibin Zhu, Lei Feng, Kaimin Yu, Yuanfang Zhang, Wen Chen, Jianzhong Hao

**Affiliations:** 1 School of Ocean Information Engineering, Jimei University, Xiamen 361021, China; peibin.zhu@jmu.edu.cn (P.Z.); 202211810001@jmu.edu.cn (L.F.); 202311810006@jmu.edu.cn (Y.Z.); 2 School of Marine Equipment and Mechanical Engineering, Jimei University, Xiamen 361021, China; 202212855006@jmu.edu.cn; 3 Institute for Infocomm Research (I2R), Agency for Science, Technology and Research (A*STAR), Singapore 138632, Singapore; haoemily@i2r.a-star.edu.sg

## Removal of an Author

In the published publication [1], Meiling Dai was included as an author by mistake. The corrected Author Contributions statement is as follows:

**Author Contributions:** Conceptualization, P.Z.; methodology, W.C. and J.H.; software, L.F.; validation, Y.Z.; formal analysis, P.Z.; investigation, K.Y.; resources, W.C.; data curation, L.F.; writing—original draft preparation, L.F.; writing—review and editing, W.C.; visualization, Y.Z.; supervision, W.C. and P.Z.; project administration, W.C. and P.Z.; funding acquisition, W.C. and P.Z. All authors have read and agreed to the published version of the manuscript.

## Affiliations Correction

Since Meiling Dai was removed from the authorship, her affiliation was also removed. Additionally, in the original publication, there was an error in the affiliation for Jianzhong Hao; the correct affiliation is as follows:

^3^ Institute for Infocomm Research (I2R), Agency for Science, Technology and Research (A*STAR), Singapore 138632, Singapore

## Addition to the Acknowledgements Section

The assistance from Meiling Dai has been included in the Acknowledgements section, as follows:

**Acknowledgments:** The authors would like to thank Meiling Dai from Jimei University for her help in reviewing the English.

The authors state that the scientific conclusions are unaffected. This correction was approved by the Academic Editor. The original publication has also been updated.
