# Supplementary material for: Enhanced DWT for Denoising Heartbeat Signal in Non-Invasive Detection
Source: Sensors (Basel). 2025 Mar 11;25(6):1743. doi: 10.3390/s25061743 (PMC11946730; doi:10.3390/s25061743)
Supplement: Supplementary file 1 [file sensors-25-01743-s001.zip › sensors-3436373-supplementary.pdf]

Supplementary Material S1

Table S1: Comparison of the output SNRs of different ECG signals with AWG noise denoised by different threshold functions.

Record	Input SNR	Enhanced DWT	Improved T + soft	Improved T + hard	Universal + soft	EMD+ DWT	VMD+ DWT	EEMD + LM	LWT	AFDA
109	-10	**3.6605**	2.3097	1.2009	1.9408	-0.8393	-0.4289	1.1242	-0.0255	3.3854
	-5	**6.7579**	6.0628	5.2651	5.6704	4.0902	4.4272	6.7399	2.9929	6.3857
	0	**10.3856**	9.9883	9.0130	8.8299	8.8432	9.1380	10.1157	6.3115	10.1177
	5	**13.9086**	13.6149	13.0410	12.9963	13.3118	13.6119	12.6477	9.7489	13.6529
	10	**17.3421**	16.5900	16.5795	16.5465	17.1546	17.2795	13.9730	12.8452	17.2236
	15	**20.6794**	19.7857	19.7282	19.7826	20.3518	20.2768	14.0731	16.8150	20.5863
	20	**24.0954**	22.9469	22.9423	22.9647	23.2157	23.2913	14.1422	20.0266	23.6792
	25	**27.8777**	27.1207	27.1092	27.1183	27.0957	26.8013	14.1358	21.8334	27.7525
	30	**30.8848**	30.6744	30.6547	30.6447	30.6323	30.1153	14.1614	22.5991	30.7628
233	-10	**3.1537**	1.9760	0.7775	1.5112	-0.8474	-0.4576	-1.8144	-0.1276	1.9037
	-5	**6.3026**	5.8862	4.6033	4.8898	4.0211	4.2976	5.6894	2.6148	5.9587
	0	**10.2960**	9.9829	9.0625	8.5220	8.7530	9.0692	9.1472	5.5788	9.6276
	5	**14.0909**	13.6960	12.9867	12.4745	13.1938	13.4483	10.9148	9.3655	13.7696
	10	**17.9231**	17.5255	16.5168	15.8700	17.1976	17.6340	11.6123	12.5361	17.7213
	15	**21.7128**	21.0441	20.8947	20.4491	21.1096	21.0324	11.8123	16.6853	21.5134
	20	**25.7245**	24.9228	24.6691	24.3749	24.7772	24.3078	11.9057	19.7031	25.1208
	25	**29.5440**	29.0664	28.2873	27.7917	27.9926	27.5072	11.9566	21.3699	29.3168
	30	**33.3108**	32.8705	32.6216	32.4078	32.3918	31.3258	11.9566	22.0625	32.5299
Cu07	-10	**2.7511**	1.2656	0.5878	0.9020	-0.8873	-0.4491	-0.7827	-0.2161	2.4525
	-5	**5.5103**	4.9585	3.8560	4.0591	3.8235	4.1236	5.2592	1.9770	5.1444
	0	**8.9570**	8.7839	7.9929	8.0813	8.2831	8.5405	7.9180	5.1776	8.5802
	5	**12.8007**	12.6691	11.7979	11.6127	12.3762	12.3406	9.1497	8.7278	12.4119
	10	**16.7140**	16.6055	16.0583	15.5241	16.1202	15.8330	9.6385	12.3064	16.6076
	15	**20.5297**	20.3209	19.8280	19.6977	19.8231	19.5503	9.6509	16.0540	20.4103
	20	**24.1049**	23.8454	22.9148	22.9014	23.1631	22.9174	9.5969	18.5374	23.9533
	25	**28.3559**	27.5534	27.5390	27.5494	27.5301	26.8313	9.6195	19.7600	27.9371
	30	**32.3823**	31.8591	31.6693	31.6731	31.6619	30.9790	9.6147	20.2332	32.2325
Cu11	-10	**3.9116**	2.9599	1.5001	2.4651	-0.8317	-0.4521	-4.5029	0.2759	3.4023
	-5	**7.1374**	6.9382	5.8574	6.3167	4.1157	4.4706	5.0364	3.3065	6.9303
	0	**10.8127**	10.6651	9.6256	10.0207	9.0193	9.2432	10.5235	7.0445	10.6273
	5	**14.6113**	14.5305	13.5949	13.6729	13.7585	13.8649	14.1707	10.2317	14.4908
	10	**18.7212**	18.4556	18.0116	18.1794	17.9597	17.8831	15.4217	13.8741	18.4629
	15	**22.6015**	22.3472	21.6424	21.6529	22.1546	21.7128	16.9947	17.3424	22.0654
	20	**26.2925**	26.2358	25.5328	25.6047	25.6071	25.2863	16.9382	21.1918	26.0481
	25	**30.1104**	29.7874	29.7441	29.7808	29.8738	29.6994	17.1090	23.7971	29.9880
	30	**33.9440**	32.9074	32.8914	32.9056	33.2181	33.5185	17.1979	25.0902	33.1013
S0016lrem	-10	**4.5947**	3.7778	2.6079	1.7452	-0.8161	-0.4162	1.8407	-0.0542	3.7376
	-5	**8.6278**	7.9380	7.0794	5.8106	4.1434	4.5340	6.3795	2.9620	8.3535
	0	**13.0196**	12.2366	11.7300	9.7137	9.0500	9.2989	10.9989	6.3094	12.4216
	5	**16.7264**	15.9433	15.4897	13.6165	13.7771	14.1420	13.0056	9.8653	15.9862
	10	**20.3794**	19.6346	18.3617	17.8979	18.3923	18.7464	14.3959	13.0212	19.7239
	15	**23.8424**	23.0508	22.2858	21.5535	22.4454	22.8538	15.0628	16.9889	23.2180
	20	**26.5903**	25.7963	25.5683	24.7607	25.8126	26.1802	15.3583	20.3593	26.1249
	25	**29.2731**	28.1932	27.9096	27.9600	28.2757	28.4827	15.3974	22.3609	28.9712

	30	**32.2831**	31.6036	31.5977	31.6011	31.5888	31.3169	15.4764	23.2569	31.9513
S0026lrem	-10	**6.1646**	4.8824	3.5983	4.3801	-0.7315	-0.3005	1.0722	1.7108	5.1725
	-5	**9.8376**	8.9681	8.0004	8.2403	4.1801	4.6387	7.1564	5.2572	9.5743
	0	**14.2582**	13.4008	12.8333	12.0199	9.2099	9.5939	11.1614	8.2397	13.7558
	5	**17.8516**	17.2776	16.5969	16.1741	14.1482	14.4713	15.7424	11.9859	17.2695
	10	**21.3149**	20.7396	20.1180	19.7457	18.9906	19.3894	17.8783	15.1890	20.8165
	15	**24.7025**	23.5245	23.0342	23.1357	23.6606	24.1987	19.5658	18.7854	24.4960
	20	**28.8401**	26.7887	26.6549	27.0285	27.9739	28.5183	20.1504	22.3017	28.7593
	25	**32.0889**	30.3504	30.3308	30.4225	31.7750	31.9613	20.4970	26.0822	31.8631
	30	**35.3614**	34.1591	34.1603	34.1873	34.6833	35.0444	20.4046	28.5619	35.0665

Table S2: Comparison of the output RMSEs of different ECG signals with AWG noise denoised by different threshold functions.

Record	Input SNR	Enhanced DWT	Improved T + soft	Improved T + hard	Universal + soft	EMD+ DWT	VMD+ DWT	EEMD + LM	LWT	AFDA
109	-10	**0.2126**	0.2487	0.2823	0.2590	0.3566	0.3402	0.2843	0.3247	0.2191
	-5	**0.1488**	0.1613	0.1767	0.1686	0.2022	0.1945	0.1490	0.2294	0.1551
	0	**0.0979**	0.1026	0.1148	0.1171	0.1170	0.1131	0.1010	0.1565	0.1009
	5	**0.0653**	0.0675	0.0722	0.0725	0.0699	0.0676	0.0755	0.1054	0.0683
	10	**0.0444**	0.0479	0.0480	0.0482	0.0449	0.0445	0.0648	0.0738	0.0445
	15	**0.0299**	0.0332	0.0334	0.0332	0.0311	0.0314	0.0640	0.0467	0.0303
	20	**0.0202**	0.0231	0.0231	0.0230	0.0224	0.0222	0.0635	0.0323	0.0212
	25	**0.0131**	0.0143	0.0143	0.0143	0.0143	0.0148	0.0636	0.0262	0.0133
	30	**0.0092**	0.0095	0.0095	0.0095	0.0095	0.0101	0.0634	0.0240	0.0094
233	-10	**0.3761**	0.4315	0.4950	0.4540	0.5956	0.5695	0.6655	0.5481	0.4338
	-5	**0.2616**	0.2748	0.3181	0.3077	0.3401	0.3294	0.2805	0.3998	0.1783
	0	**0.1652**	0.1714	0.1906	0.2025	0.1972	0.1902	0.1884	0.2842	0.2003
	5	**0.1067**	0.1117	0.1212	0.1285	0.1183	0.1149	0.1537	0.1837	0.1106
	10	**0.0686**	0.0719	0.0809	0.0869	0.0746	0.0709	0.1418	0.1275	0.0702
	15	**0.0443**	0.0479	0.0487	0.0513	0.0475	0.0480	0.1391	0.0791	0.0454
	20	**0.0279**	0.0306	0.0316	0.0326	0.0312	0.0329	0.1371	0.0559	0.0299
	25	**0.0180**	0.0190	0.0208	0.0220	0.0215	0.0228	0.1363	0.0461	0.0184
	30	**0.0117**	0.0123	0.0126	0.0129	0.0130	0.0147	0.1363	0.0426	0.0128
Cu07	-10	**0.3556**	0.4220	0.4561	0.4398	0.5405	0.5139	0.5338	0.5001	0.3677
	-5	**0.2587**	0.2759	0.3130	0.3057	0.3142	0.3035	0.2662	0.3886	0.2697
	0	**0.1740**	0.1775	0.1944	0.1924	0.1880	0.1826	0.1960	0.2688	0.1816
	5	**0.1118**	0.1135	0.1255	0.1281	0.1174	0.1179	0.1701	0.1786	0.1168
	10	**0.0712**	0.0721	0.0768	0.0817	0.0763	0.0788	0.1608	0.1183	0.0721
	15	**0.0459**	0.0470	0.0498	0.0505	0.0498	0.0514	0.1606	0.0768	0.0465
	20	**0.0304**	0.0313	0.0349	0.0349	0.0339	0.0349	0.1616	0.0577	0.0309
	25	**0.0186**	0.0204	0.0205	0.0205	0.0205	0.0222	0.1612	0.0501	0.0196
	30	**0.0117**	0.0125	0.0127	0.0127	0.0127	0.0138	0.1612	0.0475	0.0119
Cu11	-10	**0.5317**	0.5938	0.7025	0.6282	0.9180	0.8787	1.4001	0.8082	0.5634
	-5	**0.3667**	0.3752	0.4249	0.4031	0.5194	0.4985	0.4669	0.5700	0.3753
	0	**0.2402**	0.2443	0.2754	0.2631	0.2953	0.2878	0.2482	0.3706	0.2452
	5	**0.1551**	0.1566	0.1744	0.1728	0.1711	0.1690	0.1631	0.2568	0.1572
	10	**0.0966**	0.0996	0.1049	0.1028	0.1055	0.1064	0.1412	0.1688	0.0995
	15	**0.0618**	0.0637	0.0690	0.0689	0.0651	0.0685	0.1178	0.1132	0.0657
	20	**0.0404**	0.0407	0.0441	0.0437	0.0437	0.0454	0.1186	0.0727	0.0416
	25	**0.0260**	0.0270	0.0272	0.0270	0.0268	0.0273	0.1163	0.0538	0.0264
	30	**0.0167**	0.0189	0.0189	0.0189	0.0182	0.0176	0.1151	0.0464	0.0184
	-10	**0.1835**	0.2017	0.2314	0.2545	0.3416	0.3263	0.2515	0.3129	0.2021

S0016lrem	-5	**0.1153**	0.1247	0.1379	0.1593	0.1930	0.1845	0.1491	0.2211	0.1188
	0	**0.0695**	0.0761	0.0806	0.1017	0.1097	0.1066	0.0876	0.1504	0.0743
	5	**0.0453**	0.0496	0.0523	0.0648	0.0637	0.0611	0.0695	0.0998	0.0493
	10	**0.0298**	0.0324	0.0376	0.0396	0.0374	0.0359	0.0593	0.0694	0.0320
	15	**0.0200**	0.0219	0.0239	0.0260	0.0235	0.0224	0.0549	0.0440	0.0214
	20	**0.0146**	0.0160	0.0164	0.0180	0.0159	0.0153	0.0530	0.0298	0.0154
	25	**0.0107**	0.0121	0.0125	0.0124	0.0120	0.0117	0.0528	0.0237	0.0111
	30	**0.0076**	0.0082	0.0082	0.0082	0.0082	0.0084	0.0523	0.0214	0.0079
S0026lrem	-10	**0.1844**	0.2142	0.2483	0.2264	0.4073	0.3877	0.3308	0.3076	0.2062
	-5	**0.1208**	0.1334	0.1493	0.1451	0.2314	0.2195	0.1642	0.2045	0.1242
	0	**0.0726**	0.0801	0.0855	0.0938	0.1297	0.1241	0.1035	0.1450	0.0767
	5	**0.0480**	0.0512	0.0554	0.0582	0.0734	0.0708	0.0611	0.0942	0.0512
	10	**0.0322**	0.0344	0.0369	0.0385	0.0421	0.0402	0.0478	0.0651	0.0340
	15	**0.0218**	0.0250	0.0264	0.0261	0.0246	0.0231	0.0393	0.0430	0.0223
	20	**0.0135**	0.0171	0.0174	0.0167	0.0149	0.0140	0.0368	0.0287	0.0136
	25	**0.0093**	0.0114	0.0114	0.0113	0.0096	0.0094	0.0353	0.0186	0.0095
	30	**0.0064**	0.0073	0.0073	0.0073	0.0069	0.0066	0.0355	0.0140	0.0066

Table S3: The SNR improvement of the denoise signal by all methods of clean ECG signals plus baseline wander noise.

Record	Enhanced DWT	Improved T + soft	Improved T + hard	Universal + soft	EMD+ DWT	VMD+ DWT	EEMD + LM	LWT	AFDA
109	**2.2477**	1.5601	-0.3611	0.0312	-0.0001	-0.0002	0.8870	-0.0057	2.1585
233	**2.7595**	2.7056	1.3277	0.0325	0.0001	0.0021	-0.3942	-0.0181	2.3970
Cu07	**1.0991**	0.8220	-0.4932	0.0183	-0.0002	0.0001	0.3909	-0.0258	0.6537
Cu11	**2.4387**	2.1902	0.5051	0.0486	0.0002	0.0001	0.8582	-0.0221	2.1108
S0016lrem	**1.4816**	0.9514	-0.8985	0.0291	0.0021	0.0016	0.2809	-0.00381	1.0700
S0026lrem	**1.0638**	0.5262	-1.1056	0.0239	0.0021	0.0022	0.2297	-0.0021	0.8341

Table S4: The RMSE of the denoise signal by all methods of clean ECG signals plus baseline wander noise.

Record	Enhanced DWT	Improved T + soft	Improved T + hard	Universal + soft	EMD+ DWT	VMD+ DWT	EEMD + LM	LWT	AFDA
109	**0.4691**	0.5078	0.6335	0.6055	0.6077	0.6077	0.5487	0.6081	0.4739
233	**0.4423**	0.4451	0.5216	0.6054	0.6077	0.6076	0.6359	0.6090	0.4611
Cu07	**0.5355**	0.5528	0.6432	0.6064	0.6077	0.6077	0.5809	0.6095	0.5636
Cu11	**0.4589**	0.4723	0.5734	0.6043	0.6077	0.6077	0.5505	0.6093	0.4765
S0016lrem	**0.5124**	0.5447	0.6739	0.6057	0.6076	0.6076	0.5883	0.6080	0.5372
S0026lrem	**0.5377**	0.5720	0.6902	0.6060	0.6076	0.6076	0.5918	0.6079	0.5431

Table S5: The SNR improvement of the denoise signal by all methods of clean ECG signals plus electrode motion noise.

Record	Enhanced DWT	Improved T + soft	Improved T + hard	Universal + soft	EMD+ DWT	VMD+ DWT	EEMD + LM	LWT	AFDA
109	**1.6064**	1.5324	0.4223	0.1082	0.0070	-0.0010	0.8087	-0.0271	1.5401
233	**2.5207**	2.4608	1.0529	0.0968	0.0143	0.0076	-0.6381	-0.0853	1.7010
Cu07	**2.2711**	1.8773	-0.8430	0.0811	-0.0003	0.0001	-1.0951	-0.1146	2.1549
Cu11	**2.8669**	2.6220	1.6463	0.0557	0.0012	0.0007	-0.3248	-0.0862	2.7381
S0016lrem	**1.5224**	1.5177	0.7515	0.1150	0.0223	0.0488	0.1684	-0.0159	1.3238
S0026lrem	**2.3489**	1.9189	0.3277	0.0939	0.0219	0.0283	0.2707	-0.0035	0.7874

Table S6: The RMSE of the denoise signal by all methods of clean ECG signals plus electrode motion noise.

Record	Enhanced DWT	Improved T + soft	Improved T + hard	Universal + soft	EMD+ DWT	VMD+ DWT	EEMD + LM	LWT	AFDA
109	**0.2340**	0.2360	0.2682	0.2781	0.2813	0.2816	0.2565	0.2824	0.2358
233	**0.2106**	0.2121	0.2494	0.2784	0.2811	0.2813	0.3030	0.2843	0.2314
Cu07	**0.2168**	0.2268	0.3102	0.2789	0.2816	0.2815	0.3193	0.2853	0.2196
Cu11	**0.2024**	0.2082	0.2329	0.2798	0.2815	0.2815	0.2922	0.2844	0.2054
S0016lrem	**0.2363**	0.2364	0.2582	0.2778	0.2808	0.2800	0.2761	0.2821	0.2417
S0026lrem	**0.2148**	0.2257	0.2711	0.2785	0.2808	0.2806	0.2729	0.2817	0.2571

Table S7: The SNR improvement of the denoise signal by all methods of clean ECG signals plus muscle artifact noise.

Record	Enhanced DWT	Improved T + soft	Improved T + hard	Universal + soft	EMD+ DWT	VMD+ DWT	EEMD + LM	LWT	AFDA
109	**1.1419**	1.1308	0.9203	0.9101	0.6651	0.7990	1.1194	-0.0628	1.1061
233	**2.5267**	2.4983	1.1501	0.8843	0.6780	0.6229	-2.6026	-0.3449	1.8772
Cu07	**4.8414**	2.5765	0.4907	0.6255	0.3807	0.3025	-2.2132	-0.4654	3.2917
Cu11	**1.2063**	1.1132	-6.4830	0.9018	0.5653	0.4460	-4.5545	-0.3806	1.0919
S0016lrem	**7.8940**	7.6822	1.9518	0.9053	0.6977	0.7681	1.7359	-0.0587	6.8210
S0026lrem	**8.4753**	8.1198	2.5646	0.9198	0.6839	0.7213	2.5032	0.3089	6.8773

Table S8: The RMSE of the denoise signal by all methods of clean ECG signals plus muscle artifact noise.

Record	Enhanced DWT	Improved T + soft	Improved T + hard	Universal + soft	EMD+ DWT	VMD+ DWT	EEMD + LM	LWT	AFDA
109	**0.1170**	0.1171	0.1200	0.1201	0.1236	0.1217	0.1172	0.1344	0.1174
233	**0.0997**	0.1000	0.1168	0.1205	0.1234	0.1242	0.1799	0.1388	0.1074
Cu07	**0.0764**	0.0991	0.1261	0.1241	0.1277	0.1288	0.1720	0.1407	0.0913
Cu11	**0.1161**	0.1173	0.2814	0.1202	0.1250	0.1267	0.2253	0.1394	0.1176
S0016lrem	**0.0538**	0.0551	0.1065	0.1202	0.1231	0.1221	0.1092	0.1343	0.0608
S0026lrem	**0.0503**	0.0524	0.0993	0.1200	0.1233	0.1228	0.0999	0.1287	0.0604

Table S9: Comparison of the output PRDs of different ECG signals with AWG noise denoised by different threshold functions.

Record	Input SNR	Enhanced DWT	Improved T + soft	Improved T + hard	Universal + soft	EMD+ DWT	VMD+ DWT	EEMD + LM	LWT	AFDA
109	-10	**65.5174**	73.7729	82.7239	79.4200	110.799	113.533	83.1130	92.4695	67.7218
	-5	**46.2511**	49.4787	54.2029	52.1280	62.5806	67.0074	52.4447	67.0866	47.9417
	0	**30.5797**	31.5232	34.7755	36.0942	36.6937	38.4909	31.9158	48.4965	31.1970
	5	**19.9976**	20.2873	21.6930	22.0503	21.6123	22.3586	23.8497	32.0543	21.1065
	10	**14.0900**	14.9287	14.9801	15.0020	14.6609	14.1879	20.2627	22.7527	14.2315
	15	**9.2344**	10.2434	10.2254	10.2449	9.3057	10.0959	19.7443	14.4695	9.3472
	20	**6.1916**	7.1024	7.1264	7.0867	6.7916	7.5831	19.4444	10.0553	6.5469
	25	**4.0560**	4.4235	4.4224	4.4252	4.2453	4.9170	19.7087	8.1490	4.0962
	30	**2.8505**	2.9269	2.9414	2.9347	2.9324	3.1369	19.4316	7.4374	2.8964
233	-10	**69.5604**	78.7701	86.8161	82.0981	111.699	110.452	114.097	95.2986	80.3184
	-5	**48.4781**	49.9290	58.0324	56.8012	67.2390	70.2255	53.6778	67.7290	49.3575
	0	**31.2575**	32.0093	35.7565	38.0093	35.7503	39.6153	33.1014	49.1887	33.0079
	5	**20.0290**	20.7465	22.6795	23.8211	22.2411	21.7574	28.4073	33.0255	20.4888
	10	**12.9038**	13.9749	15.2144	15.9464	13.3852	14.3763	26.9192	23.2217	12.9997
	15	**8.1383**	8.7123	9.2097	9.4916	8.8211	9.2981	25.6356	14.6957	8.4010
	20	**5.2462**	5.7803	5.9449	6.0971	5.6576	6.2717	25.1866	10.4048	5.5457
	25	**3.3065**	3.4979	3.8380	4.0658	3.9783	4.9051	25.1300	8.6064	3.4210
	30	**2.1621**	2.3259	2.3532	2.4092	2.3787	2.8260	25.1480	7.9693	2.3632
Cu07	-10	**73.7387**	88.1517	94.6314	91.2324	106.577	106.891	108.812	90.4553	75.4002
	-5	**52.6124**	55.8397	63.2865	62.1998	64.5770	65.9535	53.5496	70.5117	55.3063
	0	**35.6110**	36.3384	39.2624	39.5745	36.4670	39.0169	38.9759	49.9234	37.2383
	5	**22.7579**	23.0461	25.2468	26.2661	24.8816	25.5367	34.9647	35.3996	23.9556
	10	**14.8069**	14.9615	15.8884	16.8098	15.3842	16.9269	33.3919	24.0253	14.9507
	15	**9.4732**	9.6562	10.2770	10.4213	10.6485	11.9524	32.7387	15.8470	9.5386
	20	**6.1956**	6.4392	7.1798	7.1725	6.9391	8.0327	32.9568	11.9332	6.3435
	25	**3.8364**	4.2017	4.2158	4.2073	4.2599	4.9267	32.7808	10.2965	4.0100
	30	**2.3869**	2.5291	2.5954	2.5938	2.6422	3.0980	32.9678	9.7504	2.4455
Cu11	-10	**64.4672**	70.8665	79.0562	74.8878	107.182	118.395	172.445	78.4616	67.5901
	-5	**44.9843**	45.2327	49.8370	48.6719	65.9611	64.7740	57.0661	58.6886	45.0281
	0	**28.6350**	29.2468	32.2203	31.4647	35.6022	35.6178	28.3952	42.2901	29.4193
	5	**18.3242**	18.5454	20.3414	20.3564	19.9193	22.7974	19.4664	30.4857	18.8563
	10	**11.8765**	12.1936	12.7920	12.6226	12.1670	13.3603	15.5765	20.3827	11.9359
	15	**7.5811**	7.8709	8.3734	8.3809	7.7136	8.6258	14.6405	14.0220	7.8837
	20	**4.8613**	4.9037	5.3169	5.2780	5.1826	5.6766	14.1025	8.8815	4.9842
	25	**3.0946**	3.2170	3.2297	3.2201	3.1711	3.6028	13.8508	6.5372	3.1666
	30	**2.0089**	2.2547	2.2575	2.2547	2.1634	2.3452	14.0056	5.6279	2.2128
S0016lrem	-10	**60.6405**	64.4063	74.6624	82.5814	103.222	101.622	83.8031	105.416	65.0307
	-5	**36.3420**	39.2062	42.9461	50.4828	60.3951	62.6327	47.2338	70.6385	38.2229
	0	**23.2476**	25.4351	25.9068	32.8591	35.5439	35.2235	28.9278	48.3513	23.9285
	5	**14.6746**	15.9368	16.4091	21.1437	21.0238	22.2886	21.9850	32.5517	15.8741
	10	**9.5607**	10.3603	12.1518	12.6995	11.7538	13.0705	18.9657	22.7651	10.3229
	15	**6.4556**	7.1061	7.8773	8.4023	7.2051	8.0978	18.1091	14.2270	6.9039
	20	**4.6338**	5.1034	5.2539	5.7554	5.0605	5.6092	17.1871	9.6709	4.9403
	25	**3.4561**	3.8800	4.0136	3.9990	3.9110	4.2815	16.7985	7.6482	3.5599
	30	**2.4336**	2.6538	2.6544	2.6541	2.6604	3.0023	16.7360	6.8829	2.5260
	-10	**47.2133**	52.9193	60.1662	57.5365	108.816	108.586	92.4351	80.9264	51.1283

S0026lrem	-5	**31.0641**	35.6884	38.8573	37.9102	59.0297	60.0795	42.5423	55.5071	33.2110
	0	**20.3726**	22.3551	24.5372	26.0725	34.4341	34.7472	27.9932	38.6846	20.5213
	5	**12.9862**	13.5704	14.6988	15.5036	18.9752	19.8461	18.0111	25.9881	13.6939
	10	**9.0589**	9.5269	9.9359	10.2262	10.9345	12.0763	12.4338	17.5502	9.1026
	15	**5.7889**	6.6010	7.0336	6.9340	6.9065	7.2138	10.3593	11.8625	5.9592
	20	**3.9397**	4.5754	4.6702	4.4557	4.0061	3.9649	9.5999	7.7948	3.9432
	25	**2.4662**	3.0156	3.0218	3.0014	2.6092	2.8121	9.0312	5.0479	2.5517
	30	**1.7285**	1.9635	1.9659	1.9583	1.8933	1.9563	9.4841	3.7934	1.7647

Table S10: Comparison of the output SINADs of different ECG signals with AWG noise denoised by different threshold functions.

Record	Input SNR	Enhanced DWT	Improved T + soft	Improved T + hard	Universal + soft	EMD+ DWT	VMD+ DWT	EEMD + LM	LWT	AFDA
109	-10	**3.5886**	2.4089	1.1834	1.9172	-0.9685	-1.1791	1.4392	0.4197	3.3574
	-5	**6.5916**	6.0687	5.1810	5.5571	4.0872	2.9553	6.7113	3.0969	6.3454
	0	**10.1842**	9.8270	9.1527	8.6775	8.8937	7.8141	10.1405	5.6696	10.0246
	5	**13.7578**	13.3994	12.9904	12.9068	13.0849	12.0624	12.4588	9.0494	13.3475
	10	**16.9109**	16.3538	16.3572	16.2992	16.6263	15.9449	13.5681	11.8974	16.6012
	15	**19.9362**	18.8000	18.7673	18.8016	19.9549	18.2978	14.0472	15.2819	19.5364
	20	**22.1908**	21.4621	21.4476	21.4784	22.1639	21.2145	14.1559	18.1291	21.9262
	25	**24.3293**	23.0610	23.0498	23.1155	23.4713	22.8877	14.1833	19.6527	23.3980
	30	**25.4109**	24.5853	24.5786	24.5794	25.0308	24.7023	14.1323	20.2810	24.6935
233	-10	**3.2184**	2.1129	1.0322	1.4583	-1.1973	-1.4806	1.3851	0.1375	1.7916
	-5	**6.2793**	5.9310	4.8669	4.9492	3.3216	2.7639	6.2952	3.0205	5.9219
	0	**10.0930**	9.8263	8.7750	8.3560	8.4345	7.7564	9.6579	5.4404	9.5018
	5	**13.7406**	13.4325	12.5083	12.1721	12.6545	12.2339	10.5836	8.7825	13.4688
	10	**17.2537**	16.9331	15.6830	15.3689	16.9248	15.9795	11.2458	11.4584	16.9544
	15	**20.5735**	20.2145	19.9170	19.2786	19.9100	19.1762	11.5565	15.2749	20.3814
	20	**23.8135**	23.0893	22.8726	22.7024	23.0091	20.6209	11.6086	18.0864	23.3234
	25	**26.3725**	26.0077	25.5932	25.0691	25.6144	23.5280	11.6499	19.5766	26.1441
	30	**27.6959**	27.6937	27.3361	27.0954	26.7789	25.9696	11.6764	20.1638	27.2682
Cu07	-10	**2.5787**	1.1361	0.5201	0.8487	-1.6839	-2.1042	0.3408	0.5810	2.4192
	-5	**5.3920**	4.8544	3.7953	3.9938	3.5625	3.1443	5.1461	2.5964	5.0438
	0	**8.9159**	8.5973	7.8643	7.9724	7.9220	7.5888	7.6702	5.33244	8.3972
	5	**12.4220**	12.3240	11.3345	11.2688	11.9061	11.7741	9.0242	8.1997	12.0508
	10	**16.1189**	16.0923	15.3185	14.7026	15.4011	13.6474	9.4564	11.1997	15.7404
	15	**19.4137**	19.3855	18.7332	18.5906	18.7217	17.6085	9.5906	14.6042	19.2160
	20	**21.8850**	21.3459	21.2246	21.2559	21.4612	20.4145	9.5332	16.8777	21.6561
	25	**24.4961**	24.2116	23.6749	23.6814	23.5966	22.6528	9.5575	17.9903	23.9428
	30	**25.9264**	25.5319	25.4553	25.4601	25.5526	24.9427	9.5650	18.4003	25.7056
Cu11	-10	**3.9935**	3.1421	1.5927	2.6562	-0.9245	-1.1227	0.4257	1.9345	3.2496
	-5	**7.0847**	6.8793	5.7794	6.2162	3.6237	3.4123	6.2890	4.3565	6.8703
	0	**10.7205**	10.5353	9.6450	9.9098	8.9162	8.0094	9.7846	7.0393	10.5110
	5	**14.5611**	14.4614	13.5039	13.6363	13.4429	12.6664	13.6606	9.65844	14.3707
	10	**18.3953**	18.1129	17.7601	17.8957	17.5003	16.6318	15.7520	13.0082	18.2439
	15	**22.1275**	21.8578	21.1848	21.2381	21.6624	19.7696	16.7271	15.9176	21.6474
	20	**25.4270**	24.5242	24.4576	24.4832	24.6206	23.0479	16.7875	19.6855	24.9101
	25	**28.4597**	28.0541	27.9965	28.0067	28.3378	26.9815	16.9840	22.1664	28.2649
	30	**30.6425**	30.3403	30.3222	30.3362	30.3110	29.5635	16.9935	23.3500	30.2866

S0016 lrem	-10	**4.7392**	3.9653	2.1741	1.8665	-1.0069	-1.2859	2.0430	-0.7058	3.7126
	-5	**8.7264**	7.9638	6.9975	5.6893	3.9979	3.1805	6.6815	2.6301	8.3010
	0	**13.0524**	12.2121	11.5074	9.7695	8.6501	8.2828	10.6527	5.7475	12.2817
	5	**16.6856**	15.9209	15.4747	13.5454	13.3852	12.4349	12.9988	8.9009	15.9537
	10	**20.0240**	19.3382	17.6844	17.4993	17.6235	16.1769	14.1944	11.8931	19.4286
	15	**23.2649**	22.6196	21.6662	20.9963	22.0474	20.9948	14.7763	15.6227	22.7749
	20	**25.6089**	25.0932	24.8957	23.8754	25.3968	23.5688	15.1400	18.9018	25.1661
	25	**27.6451**	27.3049	26.8209	26.7987	27.2683	26.3257	15.1020	20.8464	27.5100
	30	**29.6520**	28.7954	28.6317	28.7703	28.6645	27.6002	15.2537	21.7096	28.8097
S0026 lrem	-10	**6.0844**	4.9753	3.7632	4.4663	-1.2627	-1.3049	1.9356	1.6759	5.1695
	-5	**9.7110**	8.8684	7.5760	8.3516	4.3407	3.5694	6.8629	4.9053	9.5684
	0	**14.1156**	13.3794	12.4335	11.9791	8.9331	8.2407	10.7855	7.8857	13.7285
	5	**17.6239**	17.1285	16.5238	16.0413	13.9942	12.2476	15.1749	11.1953	17.2280
	10	**20.9906**	20.5987	19.9169	19.7788	18.8674	17.3799	17.5621	14.2865	20.7520
	15	**24.6241**	23.2810	22.9425	23.0540	23.4704	22.4824	20.3461	17.6441	24.3997
	20	**27.5696**	26.5827	26.4509	26.8479	26.7399	27.1618	19.4250	20.8717	27.2802
	25	**31.2117**	29.3946	29.3769	30.0308	31.0487	29.4760	20.0077	24.5728	30.7129
	30	**33.7658**	32.8288	32.7875	32.8245	33.1147	33.1207	20.2484	26.9428	33.6138

Table S11: Comparison of the output SNRs of different MIT-BIH ECG signals with AWG noise denoised by different threshold functions.

Record	Input SNR	Enhanced DWT	Improved T + soft	Improved T + hard	Universal + soft	EMD+ DWT	VMD+ DWT	EEMD + LM	LWT
106	-10	**2.034**	0.731	1.079	0.236	-1.119	-1.908	1.024	-0.263
	-5	**4.630**	3.290	3.195	3.084	3.170	2.596	3.909	1.203
	0	**7.977**	6.636	6.151	6.139	7.045	7.607	5.300	4.392
	5	**12.128**	10.520	10.367	10.329	11.176	11.792	5.806	7.395
	10	**15.523**	14.497	14.187	14.171	15.472	15.143	5.905	11.495
	15	**19.892**	18.288	17.780	17.782	18.440	18.942	6.010	14.439
	20	**23.624**	22.168	22.163	22.156	22.413	21.115	6.032	16.050
	25	**27.159**	26.105	25.854	25.789	26.024	25.104	6.036	16.689
	30	**30.458**	29.171	28.118	28.272	29.418	28.990	6.012	16.852
118	-10	**2.592**	1.238	0.041	0.571	-1.249	-1.346	1.177	-0.213
	-5	**5.423**	5.170	4.585	4.970	3.251	3.036	5.105	1.981
	0	**8.887**	8.546	7.541	7.654	8.249	7.591	7.437	4.874
	5	**12.391**	11.502	10.679	10.769	11.773	11.662	8.448	8.208
	10	**16.124**	15.720	14.923	14.939	15.401	15.229	8.796	12.016
	15	**19.533**	19.119	18.248	18.305	18.744	19.503	8.967	15.525
	20	**23.714**	23.159	22.682	22.682	22.708	21.303	8.970	17.676
	25	**27.562**	27.116	27.140	27.116	27.104	25.750	9.012	18.674
	30	**31.268**	30.265	30.459	30.244	30.191	29.724	9.003	19.024
210	-10	**2.892**	1.489	0.618	1.037	-1.526	-1.649	1.562	-0.030
	-5	**5.907**	5.017	4.062	4.267	3.312	2.787	5.508	2.302
	0	**9.525**	9.219	8.085	8.185	8.367	7.972	8.320	5.291
	5	**13.650**	12.546	11.963	12.065	12.699	12.172	9.452	9.034
	10	**17.222**	16.603	16.553	15.136	16.315	16.830	10.011	12.352
	15	**20.569**	20.248	19.373	19.330	20.317	20.127	10.256	16.166
	20	**24.549**	23.581	22.769	22.873	23.102	21.924	10.278	18.799
	25	**27.570**	26.667	26.659	26.667	26.734	25.679	10.234	20.104
	30	**30.799**	30.068	30.084	30.047	29.969	29.975	10.336	20.623

Table S12: Comparison of the output RMSE of different MIT-BIH ECG signals with AWG noise denoised by different threshold functions.

Record	Input SNR	Enhanced DWT	Improved T + soft	Improved T + hard	Universal + soft	EMD+ DWT	VMD+ DWT	EEMD + LM	LWT
106	-10	**0.133**	0.155	0.149	0.164	0.192	0.210	0.150	0.174
	-5	**0.099**	0.115	0.117	0.118	0.117	0.125	0.108	0.147
	0	**0.067**	0.079	0.083	0.083	0.077	0.070	0.092	0.102
	5	**0.042**	0.050	0.051	0.051	0.047	0.043	0.086	0.073
	10	**0.028**	0.032	0.033	0.033	0.028	0.029	0.085	0.046
	15	**0.017**	0.021	0.022	0.022	0.020	0.019	0.084	0.033
	20	**0.011**	0.013	0.013	0.013	0.013	0.015	0.084	0.028
	25	**0.007**	0.008	0.009	0.009	0.008	0.009	0.084	0.026
	30	**0.005**	0.006	0.007	0.007	0.006	0.006	0.084	0.026
118	-10	**0.174**	0.203	0.233	0.219	0.270	0.273	0.205	0.240
	-5	**0.125**	0.129	0.138	0.132	0.161	0.165	0.130	0.187
	0	**0.084**	0.088	0.098	0.097	0.091	0.098	0.099	0.134
	5	**0.056**	0.062	0.068	0.068	0.060	0.061	0.089	0.091
	10	**0.037**	0.038	0.042	0.042	0.040	0.041	0.085	0.059
	15	**0.025**	0.026	0.029	0.028	0.027	0.025	0.083	0.040
	20	**0.015**	0.016	0.017	0.017	0.017	0.020	0.083	0.031
	25	**0.010**	0.010	0.010	0.010	0.010	0.012	0.083	0.028
	30	**0.006**	0.007	0.007	0.007	0.007	0.008	0.083	0.027
210	-10	**0.144**	0.169	0.187	0.178	0.240	0.243	0.168	0.202
	-5	**0.102**	0.113	0.126	0.123	0.137	0.146	0.107	0.154
	0	**0.067**	0.070	0.079	0.078	0.077	0.080	0.077	0.109
	5	**0.042**	0.047	0.051	0.050	0.047	0.049	0.068	0.071
	10	**0.028**	0.030	0.030	0.035	0.030	0.029	0.063	0.049
	15	**0.019**	0.020	0.022	0.022	0.019	0.020	0.062	0.031
	20	**0.012**	0.013	0.015	0.014	0.014	0.016	0.062	0.023
	25	**0.008**	0.009	0.009	0.009	0.009	0.010	0.062	0.020
	30	**0.006**	0.006	0.006	0.006	0.006	0.006	0.061	0.019

Table S13: Comparison of the output SNRs of different CU ECG signals with AWG noise denoised by different threshold functions.

Record	Input SNR	Enhanced DWT	Improved T + soft	Improved T + hard	Universal + soft	EMD+ DWT	VMD+ DWT	EEMD + LM	LWT
Cu03	-10	**2.976**	2.501	2.114	2.380	-1.541	-1.673	1.142	0.274
	-5	**5.860**	5.837	5.554	5.837	3.125	3.033	6.132	3.065
	0	**9.603**	9.449	9.088	9.240	8.302	7.913	10.081	6.303
	5	**13.298**	12.707	12.506	12.707	12.687	12.635	12.156	9.794
	10	**17.554**	16.225	16.697	16.180	16.921	16.790	13.259	12.985
	15	**20.817**	20.182	20.230	20.183	20.410	19.979	13.517	16.916
	20	**24.443**	23.904	23.767	23.761	23.899	22.547	13.498	20.239
	25	**28.474**	27.549	27.529	27.549	27.516	26.915	13.514	22.230
	30	**31.982**	31.445	31.445	31.445	31.588	29.878	13.618	23.100
Cu18	-10	**2.976**	2.501	2.114	2.380	-1.541	-1.673	1.142	0.274
	-5	**5.860**	5.837	5.554	5.837	3.125	3.033	6.132	3.065
	0	**9.603**	9.449	9.088	9.240	8.302	7.913	10.081	6.303
	5	**13.298**	12.707	12.506	12.707	12.687	12.635	12.156	9.794
	10	**17.554**	16.225	16.697	16.180	16.921	16.790	13.259	12.985
	15	**20.817**	20.182	20.230	20.183	20.410	19.979	13.517	16.916

	20	**24.443**	23.904	23.767	23.761	23.899	22.547	13.498	20.239
	25	**28.474**	27.549	27.529	27.549	27.516	26.915	13.514	22.230
	30	**31.982**	31.445	31.445	31.445	31.588	29.878	13.618	23.100
Cu30	-10	**1.942**	1.301	1.109	1.049	-1.411	-1.635	0.833	-0.095
	-5	**4.893**	4.115	3.617	3.602	2.782	2.765	4.713	1.919
	0	**8.049**	7.296	7.212	7.283	7.745	7.646	6.360	4.597
	5	**11.335**	10.838	10.274	10.311	11.015	11.247	6.913	7.551
	10	**14.653**	14.481	13.796	13.851	14.326	14.210	6.509	11.594
	15	**19.034**	18.339	17.652	17.678	17.623	17.358	6.531	14.629
	20	**22.704**	21.971	21.845	21.868	22.046	20.898	6.725	16.323
	25	**26.928**	25.589	25.469	25.469	25.455	24.640	6.646	17.014
	30	**29.690**	28.517	27.376	27.431	27.497	27.571	6.777	17.211

Table S14: Comparison of the output RMSE of different CU ECG signals with AWG noise denoised by different threshold functions.

Record	Input SNR	Enhanced DWT	Improved T + soft	Improved T + hard	Universal + soft	EMD+ DWT	VMD+ DWT	EEMD + LM	LWT
Cu03	-10	**0.255**	0.269	0.281	0.273	0.428	0.435	0.315	0.348
	-5	**0.183**	0.183	0.189	0.183	0.250	0.253	0.177	0.252
	0	**0.119**	0.121	0.126	0.124	0.138	0.144	0.112	0.174
	5	**0.078**	0.083	0.085	0.083	0.083	0.084	0.088	0.116
	10	**0.048**	0.055	0.052	0.056	0.051	0.052	0.078	0.081
	15	**0.033**	0.035	0.035	0.035	0.034	0.036	0.076	0.051
	20	**0.022**	0.023	0.023	0.023	0.023	0.027	0.076	0.035
	25	**0.014**	0.015	0.015	0.015	0.015	0.016	0.076	0.028
	30	**0.009**	0.010	0.010	0.010	0.009	0.012	0.075	0.025
Cu18	-10	**0.189**	0.223	0.232	0.223	0.285	0.297	0.212	0.254
	-5	**0.153**	0.171	0.171	0.168	0.176	0.172	0.153	0.211
	0	**0.101**	0.103	0.108	0.106	0.106	0.107	0.125	0.145
	5	**0.061**	0.067	0.072	0.072	0.062	0.066	0.114	0.102
	10	**0.041**	0.044	0.045	0.045	0.042	0.042	0.109	0.065
	15	**0.025**	0.028	0.029	0.029	0.027	0.030	0.110	0.045
	20	**0.016**	0.018	0.018	0.018	0.018	0.022	0.110	0.037
	25	**0.010**	0.011	0.011	0.011	0.011	0.013	0.110	0.034
	30	**0.007**	0.007	0.007	0.007	0.007	0.008	0.110	0.033
Cu30	-10	**0.179**	0.193	0.197	0.198	0.263	0.270	0.203	0.226
	-5	**0.127**	0.139	0.148	0.148	0.163	0.163	0.130	0.180
	0	**0.089**	0.097	0.098	0.097	0.092	0.093	0.108	0.132
	5	**0.061**	0.064	0.069	0.068	0.063	0.061	0.101	0.094
	10	**0.041**	0.042	0.046	0.045	0.043	0.044	0.106	0.060
	15	**0.025**	0.027	0.029	0.029	0.029	0.030	0.106	0.043
	20	**0.016**	0.018	0.018	0.018	0.018	0.020	0.103	0.036
	25	**0.010**	0.012	0.012	0.012	0.012	0.013	0.104	0.033
	30	**0.007**	0.008	0.010	0.010	0.009	0.009	0.103	0.033

Table S15: Comparison of the output SNRs of different CU ECG signals with AWG noise denoised by different threshold functions.

Record	Input SNR	Enhanced DWT	Improved T + soft	Improved T + hard	Universal + soft	EMD+ DWT	VMD+ DWT	EEMD + LM	LWT
	-10	**4.908**	3.399	3.080	3.297	-1.230	-1.561	1.697	1.760
	-5	**8.625**	8.243	7.348	7.219	3.743	3.792	6.848	4.789
	0	**11.535**	11.181	10.576	10.486	8.320	8.648	10.950	7.604
	5	**14.607**	13.349	13.715	13.518	13.016	12.285	13.187	10.473

S0031lrem	10	**18.055**	16.525	16.172	16.856	17.815	17.836	15.358	14.196
	15	**21.728**	20.040	19.955	20.086	21.667	20.048	15.641	17.266
	20	**25.438**	24.478	24.369	24.375	24.736	23.833	15.752	21.102
	25	**28.272**	27.467	27.168	27.159	27.403	26.573	15.848	23.670
	30	**30.625**	30.347	30.396	30.349	30.386	28.982	15.756	24.940
S0038 lrem	-10	**5.606**	3.990	3.049	3.902	-2.278	-2.044	1.541	1.488
	-5	**10.175**	9.394	8.460	7.624	2.408	2.329	7.093	4.941
	0	**13.413**	12.497	12.879	11.081	7.596	7.764	11.255	8.045
	5	**16.555**	16.423	16.339	14.741	12.490	12.500	15.772	11.468
	10	**20.180**	18.237	18.053	18.833	17.315	17.119	18.398	14.903
	15	**23.244**	22.076	22.003	22.020	21.622	21.229	20.230	18.135
	20	**26.214**	25.154	24.876	25.207	25.203	25.278	20.605	21.718
	25	**28.947**	27.696	27.660	27.791	27.775	27.703	20.078	24.831
	30	**31.198**	30.952	30.952	30.952	30.212	30.463	20.603	26.568
S0057lrem	-10	**5.291**	3.923	3.226	3.271	-1.380	-0.945	1.878	1.151
	-5	**8.920**	6.434	5.772	6.425	3.107	4.155	6.108	3.929
	0	**12.721**	12.810	11.932	11.036	8.973	8.563	11.554	7.486
	5	**16.211**	15.774	15.283	14.020	13.554	13.422	13.789	10.418
	10	**20.040**	19.690	19.624	18.027	17.739	17.282	16.881	14.208
	15	**24.234**	23.246	22.744	21.850	22.042	22.012	18.016	17.386
	20	**25.617**	25.233	25.207	24.357	25.454	25.292	18.638	21.242
	25	**28.880**	27.204	27.204	27.492	27.544	27.657	19.014	23.836
	30	**31.087**	30.844	30.844	30.876	31.084	30.966	18.568	25.118

Table S16: Comparison of the output RMSE of different PTB ECG signals with AWG noise denoised by different threshold functions.

Record	Input SNR	Enhanced DWT	Improved T + soft	Improved T + hard	Universal + soft	EMD+ DWT	VMD+ DWT	EEMD + LM	LWT
S0031lrem	-10	**0.104**	0.124	0.128	0.125	0.211	0.219	0.150	0.149
	-5	**0.068**	0.071	0.078	0.080	0.119	0.118	0.083	0.105
	0	**0.048**	0.050	0.054	0.055	0.070	0.068	0.052	0.076
	5	**0.034**	0.039	0.038	0.039	0.041	0.044	0.040	0.055
	10	**0.023**	0.027	0.028	0.026	0.024	0.023	0.031	0.036
	15	**0.015**	0.018	0.018	0.018	0.015	0.018	0.030	0.025
	20	**0.010**	0.011	0.011	0.011	0.011	0.012	0.030	0.016
	25	**0.007**	0.008	0.008	0.008	0.008	0.009	0.029	0.012
	30	**0.005**	0.006	0.006	0.006	0.006	0.007	0.030	0.011
S0038 lrem	-10	**0.086**	0.103	0.115	0.104	0.197	0.202	0.137	0.138
	-5	**0.051**	0.055	0.062	0.068	0.115	0.120	0.072	0.093
	0	**0.035**	0.039	0.037	0.046	0.063	0.064	0.045	0.065
	5	**0.024**	0.025	0.025	0.030	0.036	0.037	0.027	0.044
	10	**0.016**	0.020	0.020	0.019	0.021	0.023	0.020	0.029
	15	**0.011**	0.013	0.013	0.013	0.013	0.014	0.016	0.020
	20	**0.008**	0.009	0.009	0.009	0.008	0.009	0.015	0.013
	25	**0.006**	0.007	0.007	0.007	0.006	0.007	0.016	0.009
	30	**0.005**	0.005	0.005	0.005	0.005	0.005	0.015	0.008
S0057lrem	-10	**0.091**	0.106	0.115	0.114	0.178	0.173	0.134	0.146
	-5	**0.060**	0.079	0.086	0.079	0.106	0.097	0.082	0.106
	0	**0.038**	0.038	0.042	0.047	0.054	0.058	0.044	0.070
	5	**0.026**	0.027	0.029	0.033	0.032	0.033	0.034	0.050
	10	**0.017**	0.017	0.017	0.021	0.020	0.022	0.024	0.032
	15	**0.010**	0.011	0.012	0.013	0.012	0.013	0.021	0.022
	20	**0.009**	0.009	0.009	0.010	0.008	0.009	0.019	0.014
	25	**0.006**	0.007	0.007	0.007	0.006	0.006	0.019	0.011
	30	**0.005**	0.005	0.005	0.005	0.004	0.005	0.020	0.009
